# Variable phenotypes and penetrance between and within different zebrafish ciliary transition zone mutants

**DOI:** 10.1242/dmm.049568

**Published:** 2022-12-19

**Authors:** Jun Wang, Holly R. Thomas, Robert G. Thompson, Stephanie C. Waldrep, Joseph Fogerty, Ping Song, Zhang Li, Yongjie Ma, Peu Santra, Jonathan D. Hoover, Nan Cher Yeo, Iain A. Drummond, Bradley K. Yoder, Jeffrey D. Amack, Brian Perkins, John M. Parant

**Affiliations:** ^1^ Department of Pharmacology and Toxicology, University of Alabama at Birmingham School of Medicine, Birmingham, AL 35294, USA; ^2^ Department of Ophthalmic Research, Cole Eye Institute, Cleveland Clinic, Cleveland, OH 44106, USA; ^3^ Department of Cell, Developmental and Integrative Biology, University of Alabama at Birmingham, AL 35294, USA; ^4^ Department of Cell and Developmental Biology, SUNY Upstate Medical University, Syracuse, NY 13210, USA; ^5^ Davis Center for Aging and Regeneration, Mount Desert Island Biological Laboratory, 159 Old Bar Harbor Road, Bar Harbor, ME 04609, USA

**Keywords:** Cilia, Transition zone, Meckel syndrome, Nephronophthisis, Joubert syndrome, Zebrafish, Penetrance, Pronephric cysts, Retinal degeneration, Scoliosis, CRISPR, Crispant

## Abstract

Meckel syndrome, nephronophthisis, Joubert syndrome and Bardet–Biedl syndrome are caused by mutations in proteins that localize to the ciliary transition zone (TZ). The phenotypically distinct syndromes suggest that these TZ proteins have differing functions. However, mutations in a single TZ gene can result in multiple syndromes, suggesting that the phenotype is influenced by modifier genes. We performed a comprehensive analysis of ten zebrafish TZ mutants, including *mks1*, *tmem216*, *tmem67*, *rpgrip1l*, *cc2d2a*, *b9d2*, *cep290*, *tctn1*, *nphp1* and *nphp4*, as well as mutants in *ift88* and *ift172.* Our data indicate that variations in phenotypes exist between different TZ mutants, supporting different tissue-specific functions of these TZ genes. Further, we observed phenotypic variations within progeny of a single TZ mutant, reminiscent of multiple disease syndromes being associated with mutations in one gene. In some mutants, the dynamics of the phenotype became complex with transitory phenotypes that are corrected over time. We also demonstrated that multiple-guide-derived CRISPR/Cas9 F0 ‘crispant’ embryos recapitulate zygotic null phenotypes, and rapidly identified ciliary phenotypes in 11 cilia-associated gene candidates (*ankfn1*, *ccdc65*, *cfap57*, *fhad1*, *nme7*, *pacrg*, *saxo2*, *c1orf194*, *ttc26*, *zmynd12* and *cfap52*)*.*

## Teaser

**Summary:** Comprehensive morphological and molecular analysis of zebrafish transition zone mutants reveals differential roles of these proteins, as well as variable phenotypes within a clutch reminiscent of variability in ciliopathy patients.

## INTRODUCTION

Cilia are microtubule-based organelles that protrude from the cell surface and perform a wide range of biological functions and processes (O'Donnell and O'Bryan, 2014; Essner et al., 2005; Bustamante-Marin and Ostrowski, 2017; Lee and Gleeson, 2011; Ringers et al., 2020; Kramer-Zucker et al., 2005; Li and Sun, 2011; Kennedy and Malicki, 2009; Goetz and Anderson, 2010). Ciliopathies result from mutations in proteins that comprise the ciliary compartments, and include Meckel syndrome [MKS; Online Mendelian Inheritance in Man (OMIM) #PS249000], nephronophthisis (NPHP; OMIM #PS256100), Joubert syndrome (JBTS; OMIM #PS213300), Bardet–Biedl syndrome (BBS; OMIM #PS209900), Leber congenital amaurosis (LCA; OMIM #PS204000), Senior–Løken syndrome (SLSN; OMIM #PS609254) and others. The most prominent clinical manifestations include, but are not limited to, early-onset phenotypes, including polydactyly and neural tube closure; adult-onset phenotypes, including retinal degeneration; mental retardation, and cystic kidneys, liver and pancreas; and obesity (Sharma et al., 2008; Mitchison and Valente, 2017). Interestingly, although the disorders are defined by distinct phenotypes, in some cases, mutations in a single gene result in multiple disorders (Mitchison and Valente, 2017; Suzuki et al., 2016; Bachmann-Gagescu et al., 2015; Szymanska et al., 2014; Barroso-Gil et al., 2021). For example, centrosomal protein 290 (CEP290) mutations are found in MKS4, NPHP6, JBTS5, LCA10 and SLSN6 patients (Rachel et al., 2012). The nature of this variable phenotypic penetrance is unclear, but it is likely a consequence of genetic modifiers (Ramsbottom et al., 2020; Masyukova et al., 2016; Cardenas-Rodriguez et al., 2021). The number of genes associated with ciliopathies is expanding; for example, the Pediatric Cardiac Genomics Consortium has recently found ciliary genes associated with chronic heart disease (Li et al., 2015; Watkins et al., 2019). Further, *in silico* interaction networks have now predicted that over 1000 genes (∼5% of all genes) are involved in ciliary function; however, most have not been functionally associated with ciliary defects (Li et al., 2004; van Dam et al., 2019, 2013). Of these genetic disorders, a subset of these genes encodes proteins that localize to a specialized domain found proximal to the base of the cilium known as the transition zone (TZ) (Horst et al., 1990). The TZ is made up of at least three multi-protein functional modules that gate the entry and exit of vesicles and membrane proteins through the cilium (van Dam et al., 2013; Horst et al., 1990; Reiter et al., 2012; Williams et al., 2011).

Some of the earliest efforts to understand the TZ utilized *Caenorhabditis elegans* to define three main modules (the ‘MKS module’, the ‘NPHP module’ and the ‘CEP290 module’) that interact and function together in the assembly and gating of molecules through the TZ (van Dam et al., 2013; Horst et al., 1990; Reiter et al., 2012; Williams et al., 2011). These studies also established a hierarchical importance of different molecules within a module based on fluorescent protein-tagged reporter analysis. For example, loss of MKS-5/RPGRIP1L results in mislocalization of MKS-1, MKS-2/TMEM216, MKS-3/TMEM67, MKS-6/CC2D2A and B9D2/MKSR-2, while loss of MKS-2 results in mislocalization of MKS-1, MKS-3 and MKS-6, but not MKS-5 or B9D2 (Huang et al., 2011; Williams et al., 2011; Roberson et al., 2015). Subsequent protein–protein interaction data in human cells confirmed several of these interactions and modules (Sang et al., 2011; Garcia-Gonzalo et al., 2011; Chih et al., 2012). However, recent studies in different mouse and human cell types indicate that the hierarchy may vary depending on the cell type (Wiegering et al., 2018). It should be noted that these molecular hierarchies do not reflect phenotypic differences in mutants. In *C. elegans*, complete loss of ciliary function only occurs with loss of a component of the MKS module and the NPHP module, suggesting that the modules function redundantly toward the same gating function (Williams et al., 2008; Yee et al., 2015). Human disorders and mouse knockouts of MKS components result in severe phenotypes, independent of NPHP loss (Norris and Grimes, 2012; Sang et al., 2011; Sharma et al., 2008), suggesting that there may be an evolutionary shift in dependence on the different modules or cell type-specific dependence on the modules. Loss of NPHP module components does result in phenotypes, for example NPHP in humans and retinal degeneration/sterility in mice, but more subtle than MKS component loss, which is typically lethal (Sharma et al., 2008). Although the TZ is viewed as ‘the gate’, variations in mouse knockout phenotypes suggest that each protein component acts as a unique gate with specificities for different molecules or by having tissue-specific roles (Lewis et al., 2019; Vierkotten et al., 2007; Garcia-Gonzalo et al., 2011). For example, in mice, although *Cc2d2a* nulls are embryonic lethal as early as embryonic day (E)11.5, *Tmem67* and *Rpgrip1l* null mice are viable at birth and die shortly after (Lewis et al., 2019; Vierkotten et al., 2007; Garcia-Gonzalo et al., 2011). Further, *Cc2d2a* and *Rpgrip1l* nulls have heterotaxia and polydactyly, whereas *Tmem67* nulls do not. Using conditional knockouts, genetic ablation of *Rpgrip1l* in adult mice results in obesity, whereas ablation of *Cc2d2a* in adults does not (Lewis et al., 2019). Note that loss of either of these genes results in pronephric cysts. A comprehensive analysis of TZ mutants and tissue-specific phenotypes would shed light onto the specificities of these proteins.

The zebrafish is an excellent model for studying human diseases, especially ciliopathies. Multiple studies have analyzed TZ proteins in zebrafish utilizing predominantly morpholino knockdown technology for rapid phenotypic analysis. These studies have described multiple ciliary dysfunction-associated phenotypes, including gastrulation defects, body curvature, hydrocephalus, pronephric cysts, eye malformations and left–right (LR) symmetry defects (Lindstrand et al., 2014; French et al., 2012; Valente et al., 2010; Leitch et al., 2008; Leightner et al., 2013; Khanna et al., 2009; Gorden et al., 2008; Bachmann-Gagescu et al., 2011; Zhao and Malicki, 2011; Sayer et al., 2006; Lessieur et al., 2019; Manning et al., 2013; Serluca et al., 2009; Bisgrove et al., 2005). Uncertainty about the true loss-of-function phenotypes for several genes exists, as these studies often report differing or contradictory phenotypes, sometimes when using the same morpholino (Lindstrand et al., 2014; Slanchev et al., 2011; Kok et al., 2015). For example, *nphp1* MO1 (https://zfin.org/) in two studies produced curved body and pronephric cysts. One study found small eyes and randomized LR heart looping asymmetry; the other study found pericardial edema and hydrocephalus (Lindstrand et al., 2014; Bisgrove et al., 2005). To address conflicting results about phenotypes associated with loss of TZ components, and to better define TZ related phenotypes, we compiled a comprehensive phenotypic analysis of zebrafish null mutants in *mks1*, *tmem216* (*mks2*), *tmem67* (*mks3*), *rpgrip1l* (*mks5*), *cc2d2a* (*mks6*), *b9d2* (*mksr2*), *cep290* (*nphp6*), *tctn1* (*jbt13*), *nphp1* and *nphp4*. We were able to identify common and unique phenotypes associated with mutations in different TZ genes. Lastly, to accelerate the analysis of the hundreds of proposed ciliary genes, we demonstrated that multiplex guide CRISPR/Cas9 F0 animals recapitulate zygotic mutant phenotypes.

## RESULTS

### Generation of ciliopathy mutants

To investigate the loss-of-function phenotypes for the ciliary TZ components in zebrafish, we generated CRISPR/Cas9-derived out-of-frame mutations in *mks1*, *tmem216*, *tmem67*, *rpgrip1l*, *cc2d2a*, *b9d2*, *cep290*, *tctn1*, *nphp1* and *nphp4* genes (Figs S1 and S2); for simplicity in the following text and figures we will denote these generated alleles as ‘−’. The IFT B complex has been established as an essential protein for ciliary retrograde trafficking and ciliary function (Haycraft et al., 2005). Therefore, as a positive control for ciliary dysfunction, we included a previously characterized *ift88* Oval (henceforth referred to as ‘–’) mutant (Tsujikawa and Malicki, 2004a,b) as well as generated a novel *ift172* mutant allele. To validate protein loss in *b9d2*, *cep290*, *ift88*, *ift172*, *nphp1*, *nphp4*, *rpgrip1l* and *tmem67* mutants, we performed western blotting of human HEK293 cells and zebrafish sibling (+/+ and +/−) and mutant (−/−) protein extracts. Of the eight antibodies used, four (anti-B9D2, anti-IFT172, anti-NPHP1 and anti-NPHP4) did not recognize the appropriate band both in human or zebrafish extracts, three (anti-CEP290, anti-RPGRIP1L and anti-TMEM67) recognized human but not zebrafish protein, and one (anti-Ift88) was able to detect both human and zebrafish protein, but not Ift88 protein in the mutant −/− extracts (Fig. S3).

### Gross embryonic phenotypes

**Fig. 1.**
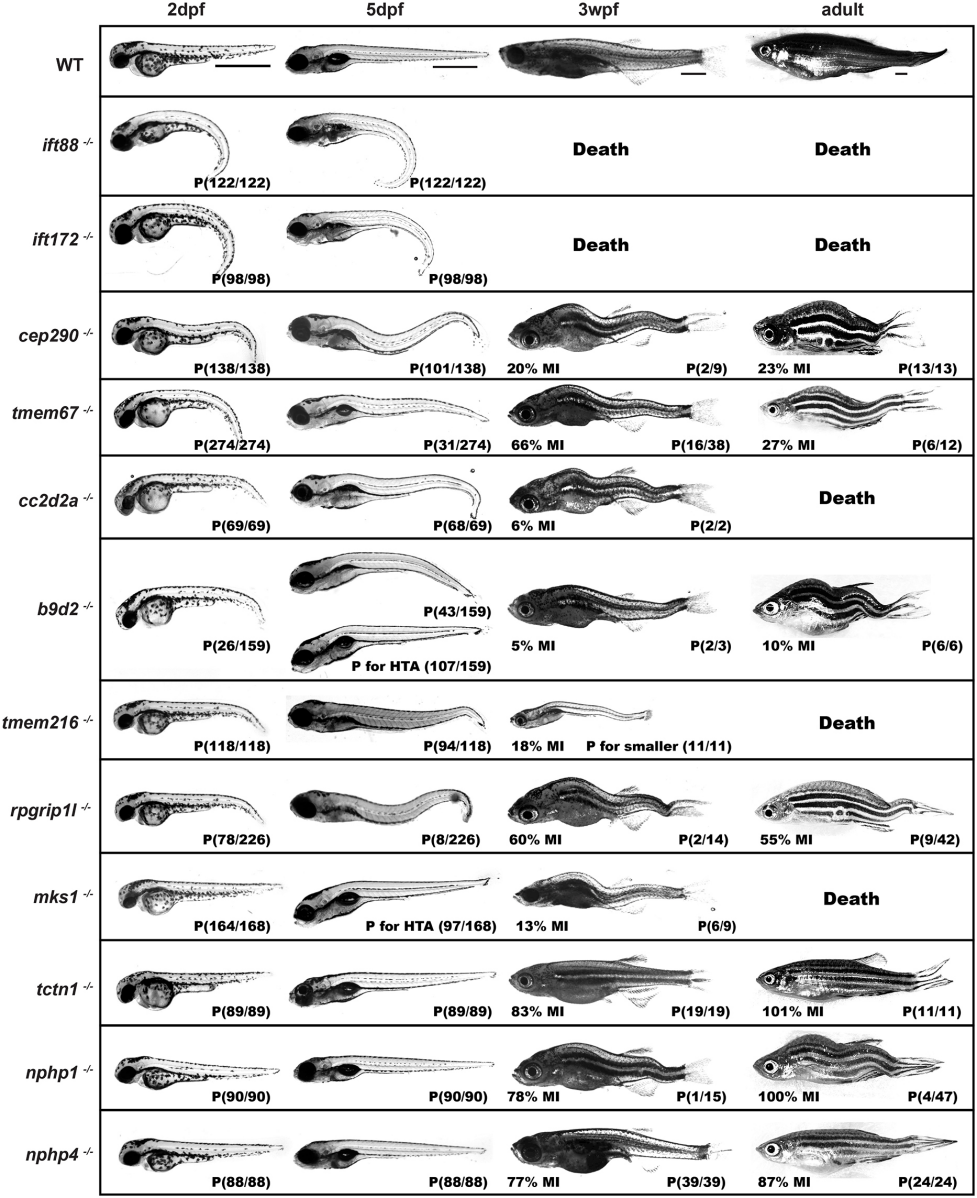
**Gross phenotype of cilia-related mutants and wild-type (WT) zebrafish across time.** Lateral views of WT and mutant animals at 2 days post fertilization (dpf), 5 dpf, 3 weeks post fertilization (wpf) and >3 months post fertilization (mpf; adult). The penetrance of the imaged phenotype [curled-down tail (CDT) and/or scoliosis for most, a decreased head-trunk-angled defect (HTA) for *mks1* and *b9d2*, and small size for *tmem216*] in −/− animals is indicated in the bottom right of each panel (P, observed/total number of −/− identified). At 3 wpf and in adults, we indicate the Mendelian inheritance (MI) in the bottom left of each panel. MI was calculated as % of the number of observed −/− divided by the expected number of −/− (1/3 of the sum of observed +/+ and +/−). Scale bars: 1000 μm.

To assess gross morphological defects of homozygous mutant embryos from these lines, we performed heterozygous×heterozygous pairwise crosses and examined embryos and larvae at 1, 2 and 5 days post fertilization (dpf). In the case of *ift88* and *ift172*, we observed a very strong curled-down tail (CDT) phenotype at both 2 dpf and 5 dpf (Fig. 1) with 100% penetrance. This is consistent with previously published *ift88* mutants (Tsujikawa and Malicki, 2004b). In *tmem216*, *tmem67*, *rpgrip1l*, *cc2d2a*, *b9d2* and *cep290*, but not *mks1*, *tctn1*, *nphp1* and *nphp4*, mutants, we observed a CDT phenotype at 2 dpf, but less severe than that of the IFT complex B mutants. Although very subtle, there was a gradient of decreasing phenotypic severity, with *cep290*>*tmem67*>*cc2d2a* and *b9d2*>*tmem216* and *rpgrip1l*; loss of *mks1*, *tctn1*, *nphp1* or *nphp4* did not result in any CDT morphological defects. To address whether the variable phenotype among mutants is due to variance in nonsense-mediated decay (NMD), we performed next-generation sequencing (NGS), and Sanger sequencing, of reverse transcription PCR (RT-PCR) products from adult heterozygote tail biopsies. The NGS and Sanger sequencing results indicate that *rpgrip1l*, *nphp4*, *cc2d2a*, *ift88*, *cep290* and *mks1* mutant alleles do not undergo NMD; *nphp1*, *tctn1*, *tmem67*, *b9d2* and *ift172* mutant alleles underwent partial NMD; and *tmem216* had complete NMD (Fig. S4), suggesting that the level of NMD does not correlate with phenotypic severity. To address whether the variability is due to maternal contributions, we performed NGS of RT-PCR products from 2 dpf and 5 dpf homozygous mutant embryos to determine the relative contribution of wild-type (WT) sequences (only derived from their heterozygous mutant mother; Fig. S5). We observed that *nphp4*, *tmem216*, *tmem67*, *cep290*, *ift88*, *mks1*, *b9d2* and *rpgrip1l* mutants had almost no contribution (<0.2%) of maternal RNA at 2 dpf, while *cc2d2a*, *ift172* and *nphp1* mutants had small contributions of maternal WT mRNA (1.92%, 11.59% and 16.48%, respectively). Importantly, the amount of maternal RNA contribution did not correlate with CDT phenotypic severity. Further, there was no correlation between the relative mRNA abundance and phenotypic severity (Fig. S6). It is important to note that we cannot address maternal protein contributions; however, homozygous inbreeding of *nphp1*^−/−^, *nphp4*^−/−^ and *tctn1*^−/−^ produced normal-looking embryos and viable adults, indicating that the lack of phenotype is not due to maternal RNA or protein. The variability in CDT phenotype among these TZ mutants indicates a differential dependence of TZ proteins regarding the severity of body axis deformities. Although *mks1* does not have a CDT phenotype, at 5 dpf, 57.7% of *msk1* mutants display a head–trunk-angled defect (HTA) (Fig. 1; Fig. S7). Further, a majority (67.3%) of *b9d2* mutants, which do not display CDT, also have this decreased HTA (Fig. 1; Fig. S7). To our knowledge, this is a novel dysfunctional cilium-associated phenotype.

### Post-embryonic phenotypes

To further assess post-embryonic phenotypes, we raised the progeny from heterozygous×heterozygous crosses. Genotyping at 3 months of age indicated normal Mendelian inheritance (MI; ∼100%) for homozygous *nphp1*, *nphp4* and *tctn1* mutant adults (Fig. 1; Fig. S8A). Although there were reduced ratios for homozygous *rpgrip1l* mutants (55%), and significantly reduced ratios for *tmem67* (27%), *cep290* (23%) and *b9d2* (10%) mutants, there was an absence of *ift88*, *ift172*, *mks1*, *tmem216* and *cc2d2a* nulls at 3 mpf (Fig. 1; Fig. S8A). To further define the time point of lethality for these mutants, we analyzed MI at 3 weeks post fertilization (wpf). For *ift88* and *ift172*, we did not identify homozygous mutants at 3 weeks of age (Fig. 1; Fig. S8B). For *mks1*, *tmem216*, *cc2d2a*, *b9d2* and *cep290* mutants, we also observed significantly reduced MI at 3 weeks (Fig. 1; Fig. S8B). For *tmem67* and *rpgripl1* mutants, there was a gradual reduction in the MI from 5 dpf to 3 months, suggesting that lethality occurs across the 3-month time point (Fig. S8D). Although lethality of *tmem216 and cc2d2a* mutants might be anticipated based on the highly penetrant 5 dpf CDT phenotype, the lethality of *mks1* mutants by 3 weeks could be associated with the decreased HTA because they lack a CDT phenotype, but there could also be another undefined cause (Fig. 1). The sub-MI in *tmem67* and *rpgrip1l* mutants is consistent with the CDT phenotypic penetrance at 5 dpf, suggesting that this is the driving cause of the lethality.

We also assessed the gross morphological phenotypes of those viable −/− animals at 3 weeks and 3 months of age (Fig. 1). We observed that four of 47 aged (>1 year) *nphp1* mutants developed a scoliotic phenotype, while none of the 24 *nphp4* mutants developed a scoliotic phenotype (Fig. 1). Note that in *nphp1 mutants*, a wavy spine could be seen in one of 15 −/− at 3 weeks, consistent with the penetrance found in the adults. This indicates that the scoliotic phenotype in *nphp1* is established between 5 dpf and 3 weeks. Although very few *tmem67*, *rpgrip1l*, *b9d2* and *cep290* homozygous nulls survived to adulthood, those that did often had a scoliosis-like phenotype. In *tmem67*, *b9d2* and *rpgrip1l* mutants, the penetrance of scoliosis at 3 months was consistent with that at 3 weeks, suggesting that the phenotype was established prior to 3 weeks of age. In contrast, in *cep290* mutants, only ∼22% of −/− showed a wavy spine at 3 weeks, while 100% had curved bodies at 3 months, suggesting that the phenotype can be acquired after 3 weeks and that *cep290* compensation is reduced after 3 wpf. We observed nine *mks1*^−/−^ at 3 weeks, the majority of which had a wavy spine; unlike other mutants, none survived to 3 months, suggesting that there are other vital processes requiring *mks1* function. We observed 11 *tmem216*^−/−^ (from two independent clutches) at 3 weeks, and, unlike other mutants, they were extremely small and did not survive to 3 months of age. This is a unique phenotype compared to the other TZ mutants and suggests that *tmem216* has a unique role in other vital biological processes. Together, these results indicate that there are different roles of TZ protein members in embryonic and adult development. In addition, these data indicate that the scoliotic phenotypes can be acquired at different developmental stages.

**Fig. 2.**
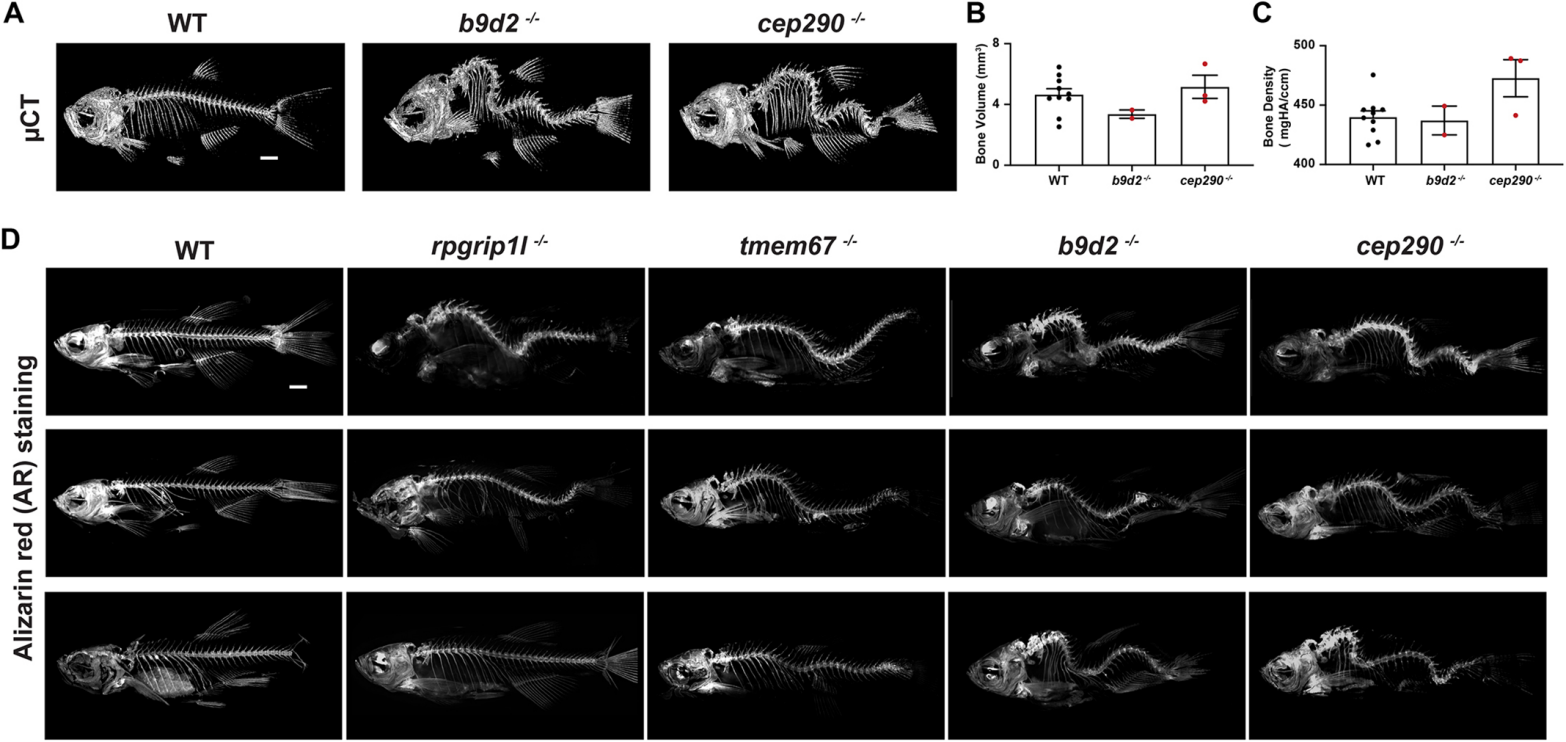
**Scoliosis in transition zone (TZ) mutants.** (A) Microcomputed tomography (μCT) images of lateral views of representative WT, and *b9d2* and *cep290* mutant zebrafish at 3 mpf. (B,C) The bone volume (B) and density (C) of WT, and *b9d2* and *cep290* mutant zebrafish. Each dot represents individual adults. WT, black dots; *b9d2*^−/−^ and *cep290*^−/−^, red dots. WT (*n*=10), *b9d2*^−/−^ (*n*=2) and *cep290*^−/−^ (*n*=3). Error bars represent ±s.e.m. (D) Alizarin Red (AR)-stained lateral view images of representative WT, and *rpgrip1l*, *tmem67*, *b9d2* and *cep290* mutant zebrafish at 3 mpf. Scale bars: 1000 μm.

To better examine the scoliotic-like phenotype, we performed microcomputed tomography (µCT) on WT, and *b9d2* and *cep290* mutant adult zebrafish (Fig. 2A). Anatomical analysis of the vertebrae suggested that they do not have any morphological defects but are oriented abnormally. Further, there was no significant difference in bone volume or bone density (Fig. 2B,C). To further evaluate other mutants, we performed Alizarin Red (AR) staining of WT, and *rpgrip1l*, *tmem67*, *b9d2* and *cep290* mutants (Fig. 2D). Consistent with µCT results, we did not find any visible defects in vertebrae. We did observe that there was a trend in phenotypic severity, such that *tmem67* and *rpgrip1l* had the least severe phenotype, whereas *b9d2* and *cep290* had the more severe phenotype.

To address fertility, breeding of homozygous males and females of *nphp1*, *nphp4*, *tctn1* and *rpgrip1l* pairs produced progeny under natural mating conditions. However, breeding of more than ten *b9d2*, *cep290* and *tmem67* homozygous males or females to WT animals did not produce progeny, suggesting that both sexes are infertile or lack breeding behaviors. The inability to breed trended with scoliotic severity, suggesting that this may be the underlying cause.

### Ciliary-dependent hair cell sensitivity to aminoglycosides

**Fig. 3.**
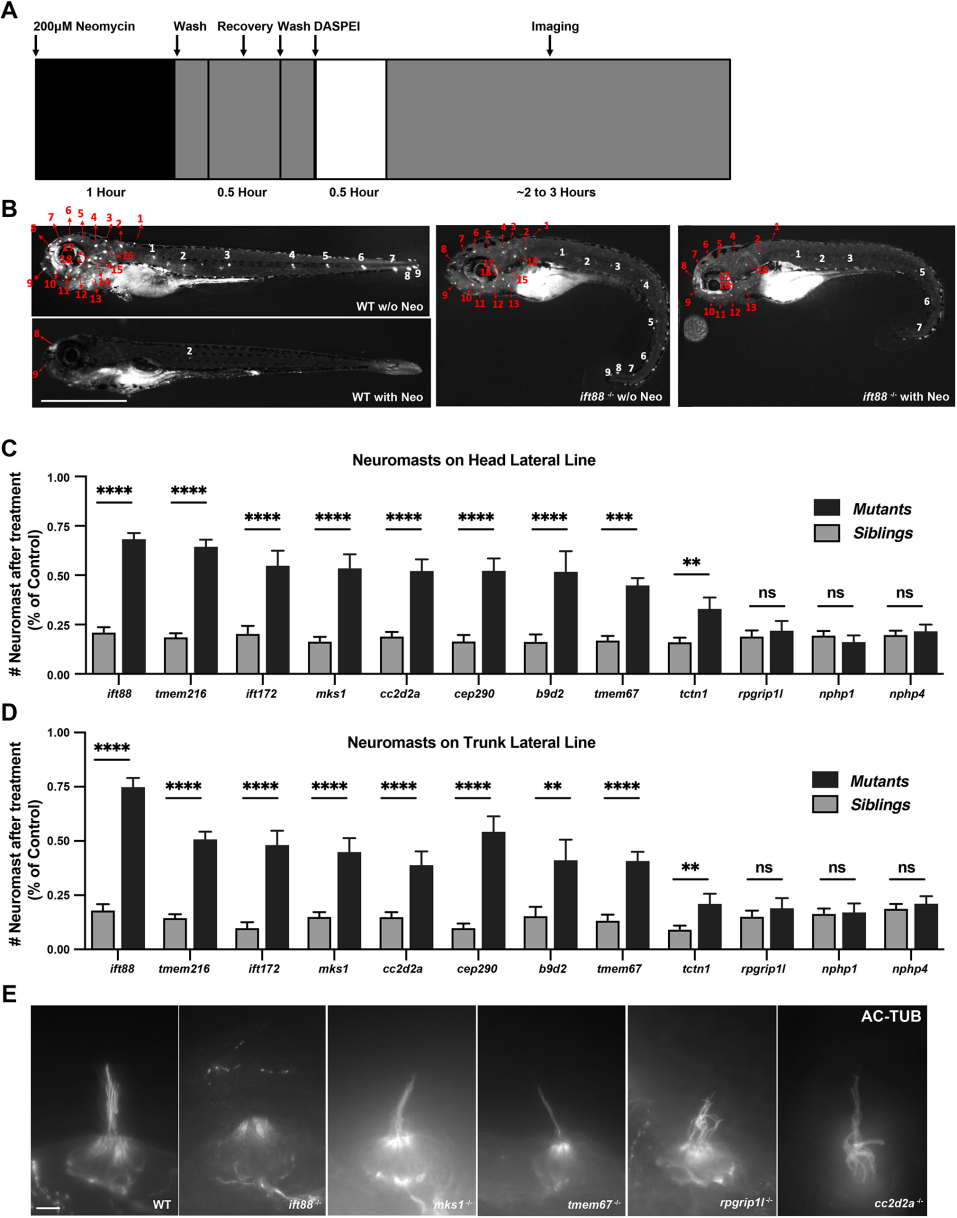
**TZ mutants have variable resistance to neomycin treatment.** (A) Diagram of timeline of neuromast staining. Zebrafish embryos at 5 dpf were exposed to 200 µM neomycin for 1 h, and neuromasts in the lateral line were stained with 2-[4-(dimethylamino) styryl]-N-ethylpyridinium iodide (DASPEI) and counted 2 h following initial neomycin exposure. (B) Representative images of neuromasts in the lateral line in WT and *ift88*^−/−^ zebrafish embryos at 5 dpf stained with DASPEI with and without (w/o) 200 nM neomycin treatment. The positions of neuromasts are numbered; red numbers indicate neuromasts in the head, and white numbers indicate neuromasts in the trunk. Scale bar: 1000 μm. (C,D) Percentage of neuromasts after neomycin treatment in the head (C) and trunk (D) lateral line for mutants and their siblings (including WT and heterozygous). Error bars represent ±s.e.m. ns, not significant; **P<0.01, ***P<0.001 and ****P<0.0001. Unpaired two-tailed Student's *t*-test (*n*>20 for all mutants from two independent experiments). (E) Representative images of anti-acetylated Tubulin staining of mechanosensory hair cells in WT, and *ift88*, *mks1*, *tmem67*, *rpgrip1l* and *cc2d2a* mutant embryos at 2 dpf. Scale bar: 5 μm.

Mechanosensory hair cells of the human inner ear are often lost in patients receiving high doses of aminoglycosides, resulting in deafness. Aminoglycosides are commonly prescribed antibiotics that are mainly used to treat aerobic gram-negative bacilli infection (Krause et al., 2016). Like humans, zebrafish have mechanosensory hair cells of the lateral line, known as neuromasts, that are sensitive to aminoglycosides (Malicki et al., 2011; Harris et al., 2003). Recent studies have indicated that dysfunctional cilia can provide resistance to aminoglycosides (Stawicki et al., 2016, 2015). Consistent with published data, loss of *ift88* resulted in resistance to neomycin and loss of kinocilia in the hair cells (Fig. 3A-E). Interestingly, we observed a differential role of TZ proteins in resistance to neomycin. In the head region, loss of *mks1*, *tmem216*, *cc2d2a*, *b9d2* and *cep290* provided a resistance to neomycin comparable to that after loss of *ift88* or *ift172*, whereas *tmem67* or *tctn1* loss provided moderate resistance, and *nphp1*, *nphp4* and *rpgrip1l* loss provided no resistance (Fig. 3C). There were slight differences in resistance between head and trunk regions, but the trend was consistent (Fig. 3C,D). At least in a subset of mutants analyzed, the differences were not due to the presence or absence of kinocilia, because *mks1*, *tmem216*, *rpgrip1l* and *cc2d2a* homozygous mutants all had kinocilia present and appeared normal (Fig. 3E), suggesting that there is a gating or trafficking defect in these mutants. Together, these data indicate that TZ proteins have differential roles or tissue-specific compensation in resistance to hair cell sensitivity to aminoglycoside treatment.

### Retinal degeneration

**Fig. 4.**
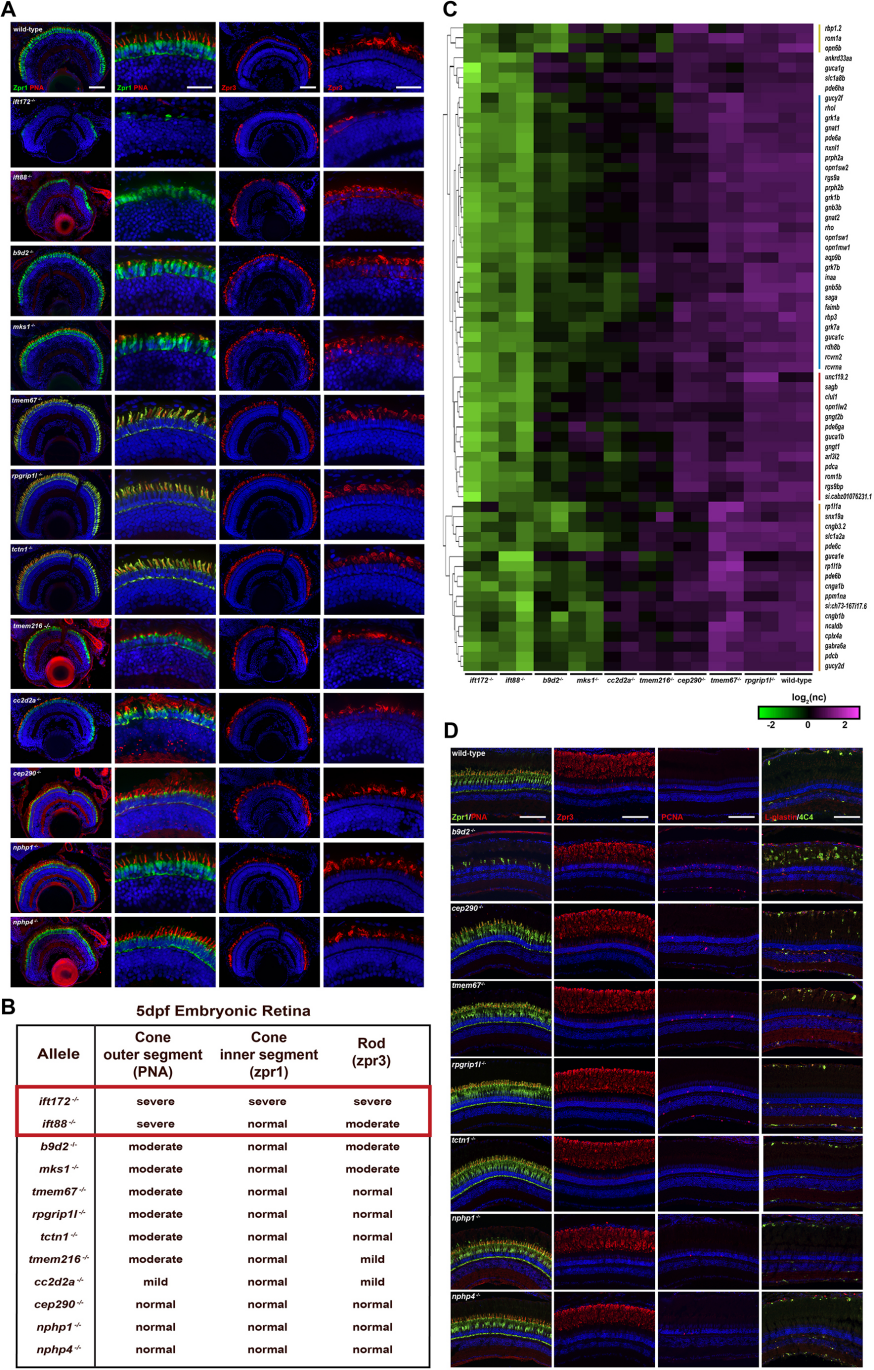
**Immunohistochemical (IHC) and transcriptional analysis of rod and cone photoreceptors for embryonic and adult retina sections of WT and cilia-related mutants.** (A) IHC staining of retinal cryosections of WT, and *ift88*, *ift172*, *b9d2*, *mks1*, *tmem67*, *rpgrip1l*, *tctn1*, *tmem216*, *cc2d2a*, *cep290*, *nphp1* and *nphp4* mutant embryos at 5 dpf. Dorsal views are shown. Images of IHC staining of cone photoreceptors with Zpr1 (green; inner segment), PNA (red; outer segment) and DAPI (blue; nuclei) in column 1. Higher-magnification images are in column 2. Images of IHC staining of rod photoreceptors with Zpr3 (red; rhodopsin – outer segment if trafficked properly) and nuclei with DAPI (blue) are in column 3. Higher-magnification images are in column 4. Scale bars: 50 µm (columns 1 and 3); 25 µm (columns 2 and 4). (B) The table depicting retinal defects observed in mutant embryos. *ift172*^−/−^ and *ift88*^−/−^ results are highlighted by a red box. (C) K-means clustering of log2 normalized counts of the 65 retinal genes. The experiment workflow for how we defined these 65 genes is shown in Fig. S9A. Colored sidebars represent different clustering groups (yellow, blue, red and orange). (D) Representative cryosectioned 3-month-old adult retinae of WT, and viable *b9d2*, *cep290*, *tmem67*, *rpgrip1l*, *tctn1*, *nphp1* and *nphp4* mutants stained with Zpr1/PNA (green/red; column 1), Zpr3 (red; column 2), Pcna (proliferating cells; red; column 3) and L-plastin/4C4 (green; pan-leukocyte marker/red; microglia marker; column 4). Scale bars: 100 µM.

Human ciliopathies often include retinal degeneration (Malicki et al., 2011; Kennedy and Malicki, 2009; Tsujikawa and Malicki, 2004a; Rachel et al., 2012). The photoreceptor cells of the retina contain an outer segment, which is a highly modified cilium. Based on Zpr1 (cone inner segments) and peanut agglutinin (PNA; cone outer segments) staining (Fig. 4A,B), loss of *ift172* resulted in the most severe defects, with loss of cone inner and outer segments. Loss of *ift88* was also severe, with complete absence of outer segments but some remaining inner segments. Loss of *mks1*, *rpgrip1l*, *tmem67*, *tmem216*, *tctn1* and *b9d2* appeared to result in strong disruption of the cone outer segments, whereas loss of *cc2d2a* resulted in mild disruption. *nphp1*, *nphp4* and *cep290* mutants had no defects at 5 dpf. This is consistent with published data on *ift88* and *cep290* mutants (Tsujikawa and Malicki, 2004b; Lessieur et al., 2019; Sukumaran and Perkins, 2009). Based on Zpr3 (rod outer segments) staining, *ift172* mutants had a clear loss of rod cells in the older central region of the retina, whereas the peripheral margin, where new rod cells are generated, had not yet degenerated (Fig. 4A,B). *ift88* mutants did not have as strong degeneration phenotype at this time point, but displayed disorganized expression of Zpr3, suggesting mislocalization of rhodopsin due to a ciliary trafficking defect. *b9d2* and *mks1* mutants also showed disorganized expression of Zpr3 and cellularity of the inner segment**,** while *tmem216* and *cc2d2a* mutants had disorganized inner segment. *nphp1*, *nphp4*, *cep290*, *tmem67*, *tctn1* and *rpgrip1l* mutants displayed normal Zpr3 staining. Together, these data indicate that loss of TZ proteins differentially contributes to early retinal degeneration in larvae. Adult 3-month-old *b9d2* mutants had considerable cone photoreceptor loss (Zpr1/PNA), a large increase in proliferation in the outer nuclear layer, where the rod precursors reside (Pcna labeling), and considerable inflammation, as indicated by the activated microglia (L-plastin/4C4) (Fig. 4D). However, adult 3-month-old *cep290*, *tmem67*, *rpgripl1*, *tctn1*, *nphp1* and *nphp4* mutants did not have any rod or cone photoreceptor degeneration. *cep290*, *tmem67* and *rpgripl1* mutants had a mild increase in proliferative cells in the outer nuclear layer and inflammation, as indicated by the activated microglia, suggesting a mild degeneration occurring (Fig. 4A,B). Interestingly, *b9d2* null mutants had embryonic rod and cone defects; however, adults had cone, but not rod, defects. This suggests there may be compensation or temporal differences for the role of *b9d2* in rods versus cones.

To define retina degenerative markers, we performed transcriptional analysis of mRNA from different 5 dpf TZ mutant embryos with retina degeneration. Among 260 conserved genes between *ift88*^−/−^ versus *ift88*^+/+^ and *ift172*^−/−^ versus *ift172*^+/+^at 5 dpf (these mutants had the strongest retinal degeneration), we defined 65 retinal-associated differentially expressed genes (DEGs) (Fig. 4C; Fig. S9, Table S4). Hierarchal clustering accurately depicted the severity of the retinal degeneration among the TZ mutants (Fig. 4C). The most prominent cluster grouped *ift88*, *ift172*, *b9d2*, *mks1* and *cc2d2a* mutants together (Fig. 4C; cluster labeled with blue sidebar). There was an *ift88*, *ift172*, *b9d2*, *mks1*, *cc2d2a* and *tmem216* mutant signature (Fig. 4C; yellow cluster), but it only contained three genes amongst the 65 conserved DEGs. There was also an *ift88*, *ift172*, *b9d2* and *mks1* mutant signature (Fig. 4C; orange cluster) and an *ift88*, *ift172* and *b9d2* mutant signature (Fig. 4C; red cluster). These clusters are likely to be indicative of phenotypic severity, with the genes contained in the blue cluster signature being the most sensitive to retinal degeneration. Note that this analysis also suggests some transcriptional differences between mutants that appear histologically similar, which will help further define the underlying mechanisms leading to retina degeneration. Note that the differences in the clusters do not denote loss of particular rod or cone cell types; for example, the blue cluster contains rod markers such as *rho*, *grk1* and *pde6a*, while the orange cluster also contains rod markers *pde6b* and *cngb1b*, and the red cluster contains rod markers *sagb*, *pde6ga* and *gngt1* as well. These likely represent differences in transcriptional sensitivity to rod dysfunction.

### LR heart looping or glomerular cyst

**Fig. 5.**
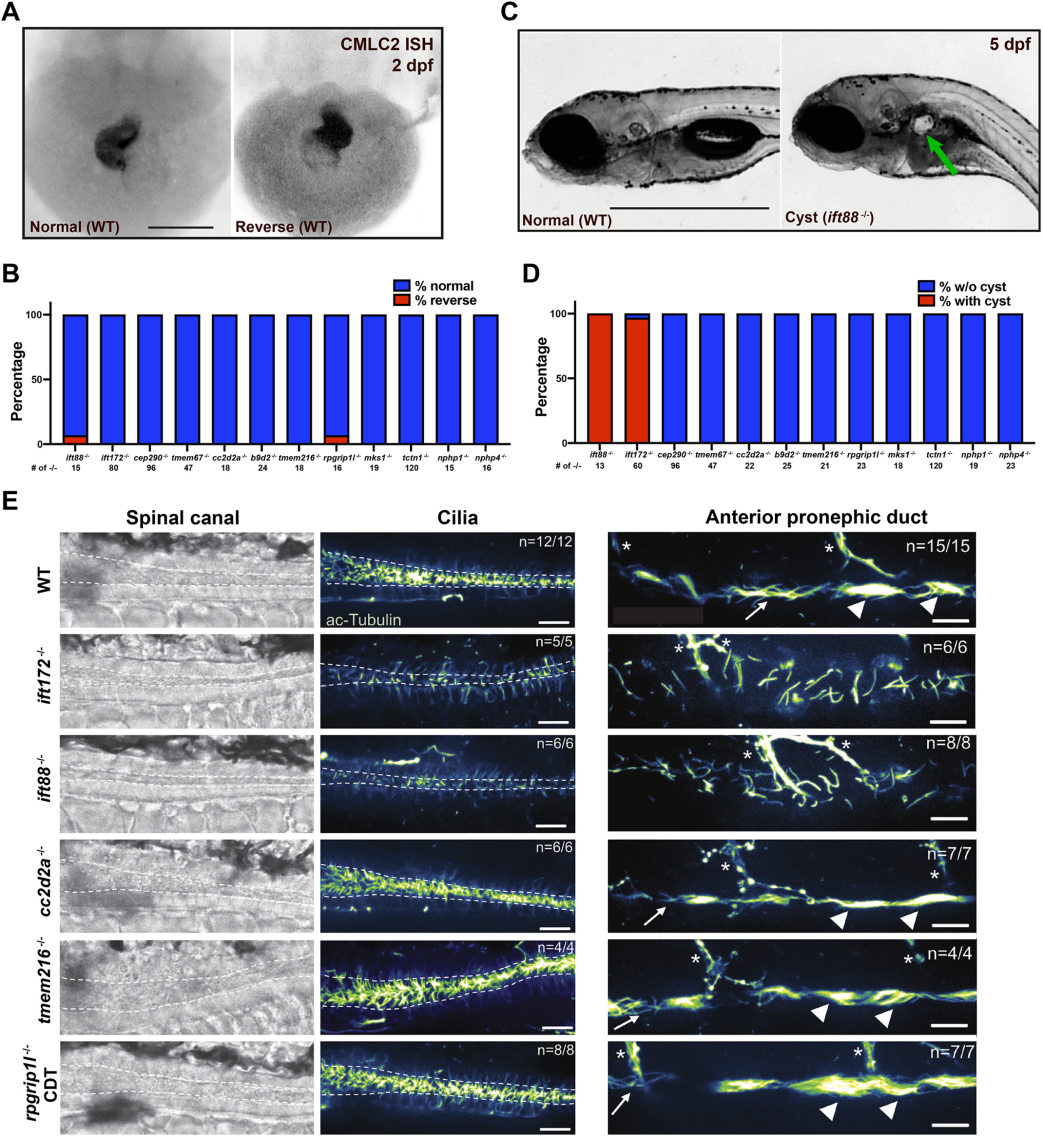
**TZ mutants lack left–right heart looping or glomerular cyst, and have normal-appearing cilia.** (A) Whole-mount *in situ* hybridization (ISH) against *cmlc2* RNA in WT zebrafish embryos at 2 dpf with normal phenotype of heart looping (left) and reverse heart looping (right). Reverse example found in WT clutch. Scale bar: 25 μm. (B) Bar graph depicting the ratio of mutant embryos with normal heart looping (% normal, blue) and with reverse heart looping (% reverse, red) at 2 dpf. The total number of each mutant is indicated. (C) Gross images of lateral views of representative zebrafish embryos with cyst (right; imaging with *ift88*^−/−^) or without cysts (left; imaging with WT embryo). Green arrow indicates the pronephric cyst. Scale bar: 1000 μm. (D) Bar graph depicting the ratio of mutant embryos without cysts (% w/o cyst, blue) and with cysts (% with cyst, red) at 5 dpf. The total number of each mutant is indicated. (E) Immunostaining of cilia in spinal canal and anterior pronephric duct. Acetylated-Tubulin antibodies were used to detect cilia in the caudal end of the spinal canal near the tip of the tail and in the anterior pronephric duct at 2 dpf. Representative brightfield images of the spinal canal, and fluorescent images of the spinal canal and anterior pronephric duct (green fire blue LUT) are shown for WT, *ift172^−^*^/−^, *ift88*^−/−^, *cc2d2a*^−/−^, *tmem216*^−/−^ and *rpgrip1l*^−/−^ embryos. WT siblings were analyzed for each mutant. All mutant embryos analyzed had a CDT phenotype. Dashed lines outline approximate boundaries of the spinal canal. The anterior pronephric duct has a mixture of multi-ciliated tufts (arrowheads) and monocilia (arrows). Motor axons are also stained by acetylated-Tubulin antibodies (marked with asterisks). *n*=number of embryos with the depicted staining/total number of embryos analyzed. Scale bars: 25 µm.

Defective LR asymmetry and pronephric cysts have been described in multiple cilia morphants (Drummond et al., 1998; O'Donnell and O'Bryan, 2014; Essner et al., 2005). LR axis symmetry defects are most apparent in the LR looping of the heart. We have observed that all the cilia-related mutants, including *ift172* and *ift88* mutants, do not have defects in LR asymmetry of the heart (Fig. 5A,B). Consistent with published data (Sun et al., 2004; Ryan et al., 2013), *ift88* and *ift172* mutants displayed cyst formation as early as 3 dpf (Fig. 5C,D). However, we did not observe cyst formation throughout the first 7 days in any of our TZ mutants (Fig. 5D). In addition, we also did not observe LR heart looping defects or pronephric cysts in progeny from homozygous mutants of *nphp1*, *nphp4*, *tctn1* or *rpgrip1l* intercrosses, suggesting that maternal *nphp1*, *nphp4*, *tctn1* or *rpgrip1l* transcripts or protein do not contribute to rescuing heart looping defects or cyst formation. These data suggest that defects in the TZ are not associated with LR axis formation or embryonic pronephric cyst formation in zebrafish.

### Cilia phenotypes

In zebrafish, the pronephros is made up of paired nephrons on each side of the body axis with two populations of ciliated cells: single ciliated principal cells and multi-ciliated cells (MCCs) (Liu et al., 2007; Malicki et al., 2011; Ma and Jiang, 2007). Surprisingly, IFT and TZ mutants have cilia that appear phenotypically normal; however, in the anterior pronephric duct, we observed a lack of multi-ciliated bundles or tufts in the *ift88* and *ift172* mutants, but not in our TZ mutants (*nphp1*, *nphp4*, *mks1*, *tmem216*, *rpgrip1l*, *cc2d2a* and *b9d2*; Fig. 5E; Fig. S10). These data suggest that loss of multi-ciliated tufts correlates with the formation of pronephric cysts. The motile cilia in the neural tube are important for movement of spinal fluid, and reduced flow is suggested to create the CDT phenotype (Grimes et al., 2016). Whereas *ift88* and *ift172* mutants had a clear reduction in ciliated cel­ls in the neural tube, *cc2d2a*, *tmem216* and *rpgrip1l* mutants did not display any discernable defect in their cilia (Fig. 5E). One possibility to explain the CDT phenotype is that although the cilia look normal, they lack motility. Therefore, we imaged cilia motility in the neural tube of *cc2d2a* mutants and WT siblings at 2 dpf. Surprisingly, we did not observe any cilia motility defect (Movies 1 and 2). Together, these data suggest that the TZ mutants do not have defect in the formation of cilia or cilia motility, and the phenotypes may stem from lack of gating of phenotype-associated molecules.

### Variable penetrance in cilia mutants

**Fig. 6.**
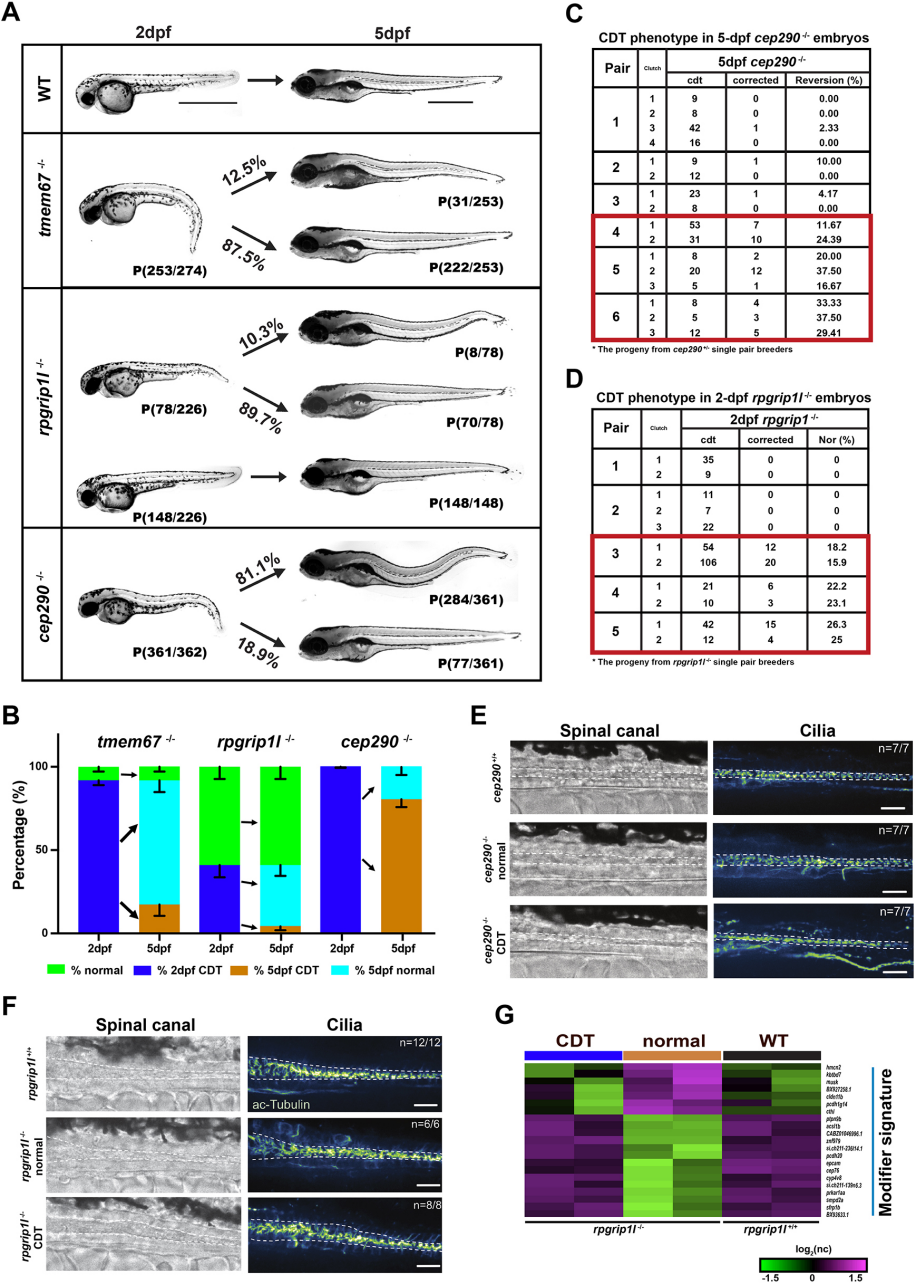
**Variable penetrance of the CDT phenotype in *tmem67*, *rpgrip1l* and *cep290* mutants.** (A) Lateral views of WT and mutants at 2 dpf and 5 dpf. All homozygous mutant embryos were obtained from a single pair of heterozygous mutant crosses that were dechorionated at 1 dpf. Embryos were sorted for CDT at 2 dpf and then reanalyzed at 5 dpf. All embryos were genotyped after phenotype analysis at 5 dpf. Arrows denote the phenotypic changes across time. Percentage was calculated as the % of observed number of −/− at 5 dpf divided by the observed number of −/− at 2 dpf. Scale bars: 1000 μm. (B) Bar graph representing distribution of CDT phenotype at 2 dpf and 5 dpf. For *tmem67*^−/−^, *n*=274 from ten breeding pairs of *tmem67*^+/−^ adults. For *cep290*^−/−^, *n*=351 from 23 breeding pairs of *cep290*^+/−^ adults. For *rpgrip1l*^−/−^, *n*=226 from ten breeding pairs of *rpgrip1l*^+/−^ adults. Arrows point to derivates of the 2 dpf phenotype. Error bars represent ±s.e.m. (C) Table depicting the revertant rate from repeatedly bred *cep290^+/−^* pairs. (D) Table depicting the ratio of embryos with normal-looking tail from repeatedly bred *rpgrip1l*^−/−^ pairs. As above, embryos were dechorionated at 1 dpf, sorted at 2 dpf, reanalyzed at 5 dpf and genotyped after. Data are only for *cep290*^−/−^ and *rpgrip1l*^−/−^ embryos. Red boxes in C and D denote pairs that had repeatedly high reversion rates. (E) Comparison of spinal canal cilia in tails of *cep290l*^−/−^ embryos with a CDT or reverted normal-looking tail at 5 dpf. Representative brightfield images of the spinal canal and fluorescent images of acetylated-Tubulin staining (green fire blue LUT) are shown for WT, *cep290^−^*^/−^ embryos with a normal-looking tail and *cep290^−^*^/−^ embryos with CDT. Dashed lines outline approximate boundaries of the spinal canal. Scale bars: 25 μm. (F) Comparison of spinal canal cilia in tails of *rpgrip1l*^−/−^ embryos with CDT or normal-looking tail. Representative brightfield images of the spinal canal and fluorescent images of acetylated-Tubulin staining (green fire blue LUT) are shown for *rpgrip1l*^+/+^ and *rpgrip1l*^−/−^ embryos with a normal-looking (straight) tail, and *rpgrip1l*^−/−^ embryos with a CDT. Dashed lines outline approximate boundaries of the spinal canal. *n*=number of embryos with the depicted staining/total number of embryos analyzed. Scale bars: 25 μm. (G) K-means clustering of log2 normalized counts define modifier signature cluster among the top 100 DEGs in 2 dpf *rpgrip1l*^−/−^ with CDT, *rpgrip1l*^−/−^ with normal-looking tail (normal) and *rpgrip1l*^+/+^ groups (WT).

Similar to the phenotypic variability in some human ciliopathy disorders, we observed variations in penetrance of CDT in several of the mutant lines. *cep290* (73%), *b9d2* (35%), *tmem67* (11%) and *rpgrip1l* (7%) mutants all had a variable penetrance of the CDT phenotype at 5 dpf (Fig. 1). In the case of the *cep290* mutant, this has been described in two separate publications with two different mutant alleles, indicating that the penetrance difference is not associated with our particular allele but with inactivation of the *cep290* gene (Lessieur et al., 2019; Cardenas-Rodriguez et al., 2021). Interestingly, almost all *tmem67^−/−^* embryos (92.3%) had a CDT at 2 dpf, but the majority of these corrected the tail to WT appearance at 5 dpf (Fig. 6A,B). *rpgrip1l^−/−^* was similar but had less CDT at 2 dpf (34.5%), and most of these corrected to a normal tail at 5 dpf (Fig. 6A,B). Almost all *cep290* mutants had CDT at 2 dpf (99.7%), but a small percentage corrected the tail at 5 dpf (Fig. 6A,B). Note that among *cep290*, *tmem67* and *rpgrip1l* mutants, all WT-looking embryos at 2 dpf maintained a normal tail morphology at 5 dpf. Together, these data suggest that, in *tmem67*, *cep290* or *rpgrip1l* mutants, there are redundant TZ genes or modifier genes that compensate after 2 dpf. We also analyzed phenotypic penetrance in the *b9d2* mutants and found the opposite consequence. All *b9d2* mutants with CDT at 2 dpf had CDT at 5 dpf, but many of the normal-tail mutants at 2 dpf converted to a CDT at 5 dpf, suggesting potential compensation early, but not after 2 dpf, in *b9d2* mutants (Fig. S7B). To further define whether this compensation in *cep290*^−/−^ is genetic or stochastic, we repeatedly bred the same pair of *cep290* heterozygous mutant fish and recorded the frequency of corrected embryos per clutch across multiple breedings. The progeny from pairs 1 to 3 consistently produced embryos that did not correct the CDT (<10%) over multiple breeding occasions, while in pairs 4 to 6, we consistently observed >20% CDT correction from breeding the same pair multiple times (Fig. 6C), indicating a genetic trend. Further, the ∼25% revertant frequency in pairs 4-6 suggests a recessive modifier gene that acts after 2 dpf. We performed a similar experiment with the *rpgrip1l* mutants. Consistent with *cep290*, the data suggest that the CDT versus normal phenotype is genetic (Fig. 6D). To determine whether there was a difference in the appearance of cilia in the neural tube between *cep290^−/−^* 5 dpf CDT and corrected embryos or *rpgrip1l^−/−^* 2 dpf CDT or normal embryos, we stained embryos with acetylated-Tubulin antibody. We did not observe differences in the cilia between 5 dpf *cep290*^−/−^ embryos with CDT or corrected tail morphology (Fig. 6E), or the cilia between the 2 dpf *rpgripl1*^−/−^ embryos with CDT or normal-looking tail morphology (Fig. 6F). Previous studies have defined modifier signatures for TZ mutants (Cardenas-Rodriguez et al., 2021; Ramsbottom et al., 2020). Therefore, we wanted to determine whether similar signatures were identified in our mutants. The differential phenotypes in *rpgrip1l*^−/−^ siblings within the same clutch provided an opportunity to define a transcriptional signature of the CDT and potential modifiers. Therefore, to examine altered gene expression underlying the CDT phenotype, RNA-sequencing (RNA-seq) analysis was performed on 2 dpf *rpgripl1*^−/−^ with CDT, *rpgripl1*^−/−^ with normal-looking tail (normal) and *rpgripl1*^+/+^ (WT). We identified 166 DEGs between 2 dpf *rpgripl1*^−/−^ with CDT and *rpgripl1*^−/−^ with normal-appearing tail (Fig. S11A, Table S5). The top 100 DEGs were analyzed (Fig. 6G; Fig. S11C) and classified into two clusters, CDT and modifier signatures (Fig. 6G), based on whether the DEG was only altered in CDT data or only in normal data, respectively, when compared to WT data. Gene ontology (GO) analysis revealed that CDT and modifier signatures include C2H2 zinc finger proteins and genes involved in nucleosome assembly, suggestive of a transcriptional change associated with correction of CDT (Fig. S11B). Together, these data indicate that there are genetic modifiers of cilia-related phenotypes in some zebrafish TZ mutants that could model human ciliopathy variability.

### CRISPR/Cas9 F0 phenotyping

**Fig. 7.**
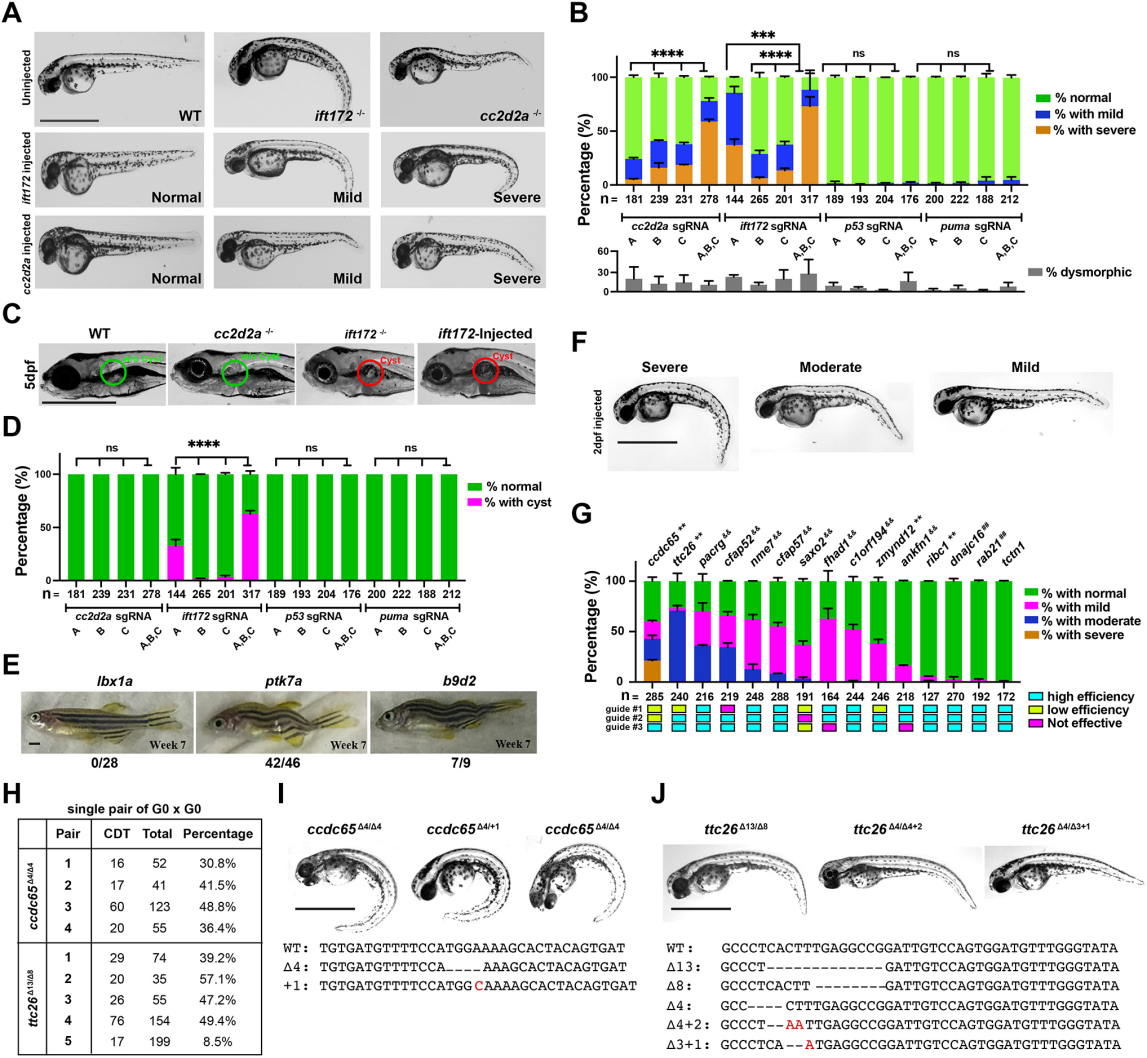
**Recapitulation of null phenotypes in G0 embryos by three-guide targeting of *ift172* and *cc2d2a*.** (A) Lateral view images of 2 dpf zygotic WT, *cc2d2a*^−/−^ and *ift172*^−/−^ embryos, as well as representative images of observed *cc2d2a* and *ift172* CRISPR G0 ‘normal’, ‘mild’ and ‘severe’ CDT embryos in each group. (B) Quantification of CDT phenotypes at 2 dpf after injections of single- or three-guide Cas9 RNPs against *cc2d2a*, *ift172*, *p53* and *puma.* Embryos were first sorted for dysmorphic phenotypes (gray, lower panel) at 1 dpf. Non-dysmorphic embryos were then scored and counted at 2 dpf for the degree of CDT phenotype as defined in A. The percentage with normal tail (green), or mild (blue) or severe (orange) CDT is indicated. ns, not significant; ***P<0.001, ****P<0.0001. Fisher's exact test for severe versus other phenotype. The *n* numbers in each group shown are from at least two independent experiments. Error bars represent ±s.e.m. (C) Representative lateral views of 5 dpf zygotic WT, *cc2d2a*^−/−^ and *ift172*^−/−^, and an example of an injected embryo at 5 dpf with pronephric cyst. (D) Quantification of cysts at 5 dpf after injections of single- or three-guide Cas9 RNPs against *cc2d2a*, *ift172*, *p53*, *puma* and *tctn1.* The percentage with (pink) or without (green) pronephric cyst formation is indicated. ns, not significant; ****P<0.0001. Fisher's exact test for cysts versus no cysts. The *n* numbers in each group shown are from at least two independent experiments. Error bars represent ±s.e.m. (E) Representative images of the lateral view of WT adult zebrafish at 7 wpf injected with four *lbx1a* gRNAs as negative controls, four-guide Cas9 RNP targeting *ptk7a* as positive controls or *b9d2*. Numbers of observed over analyzed are noted. (F) Representative images of observed 2 dpf CRISPR-injected ‘severe’, ‘moderate’ and ‘mild’ G0 embryos for each gene of interest. (G) Quantification of CDT phenotypes at 2 dpf after injection of three-guide Cas9 RNPs against 15 novel cilia gene candidates and *tctn1*. The percentage with normal tail (green), and mild (magenta), moderate (blue) or severe (orange) CDT is indicated. The *n* numbers in each group shown are from at least two independent experiments. Error bars represent ±s.e.m. The gRNA efficiency was determined by high-resolution melting curve analysis and color-labeled with blue (high efficiency), yellow (low efficiency) and magenta (without efficiency). The source of these novel genes is indicated: **genes from CiliaCarta top 50 genes; ^&&^genes from Patir et al. (2020); ^##^genes from Reiter proteomic analysis. (H) Table depicting the number of 2 dpf F1 embryos with different phenotypes from single-pair breedings of injected *ccdc65* or *ttc26* G0s. (I,J) Representative gross images of 2 dpf embryos with *ccdc65* (I) and *ttc26* (J) compound mutations. Mutations of the embryos with CDT from the two injected clutches were validated by Sanger sequencing. Sequencing results are listed comparing with WT. ‘–’ indicates the deletion. Nucleotides in red indicate the deletion. Scale bars: 1000 μm.

With the increasing number of cilia-associated disease genes needing to be functionally analyzed, and concerns over accuracy of morpholino-derived phenotypes, we pursued whether CRISPR/Cas9 F0 crispants could be used to access ciliary phenotypes. To test this concept, we first designed three guides targeting *cc2d2a* and *ift172*. Note that both zygotic null embryos have CDT phenotype, but only *ift172* nulls form pronephric cysts (Fig. 7A,C). As a control, we designed three guides targeting *p53* (*tp53*) and *puma* (*bbc3*). The zygotic *p53* and *puma* null embryos and adults do not have morphological phenotypes (Wang et al., 2021). We observed strong variability in phenotypes with single-guide injections. However, Cas9 protein injected with a three-guide combination produced higher penetrance, and the majority of embryos had a phenotype consistent with the zygotic null phenotype, suggesting production of a higher number of animals that were high chimeric nulls (Fig. 7A,B). Also, the *ift172* guides produced embryos with pronephric cysts, whereas *cc2d2a* guides did not (Fig. 7C,D). Importantly *p53* and *puma* guides produced almost no embryos with a curved body (<5%) and none with pronephric cysts. To address the post-embryonic phenotypes, we designed four guides targeting *b9d2*, *ptk7a* (positive control, previously described as a scoliotic mutant) (Hayes et al., 2014) and *lbx1a* as a negative control. By 7 wpf, we observed scoliotic phenotypes in both the *b9d2* and *ptk7a* crispants, but not the *lbx1a* crispants (Fig. 7E). Reduced viability of *b9d2* crispants was consistent with that observed in the zygotic nulls. Together, these data suggest that multi-guide-injected CRISPR/Cas9 F0s recapitulate both embryonic and juvenile cilia-associated phenotypes.

With the success of the crispant pilot experiments, we wanted to determine whether this approach could be used to define whether loss of putative cilia genes drives a ciliary CDT phenotype. Therefore, we selected 14 putative cilia genes [*ankfn1*, *cfap57*, *fhad1*, *c1orf194* (*si:ch211-163l21.7*), *pacrg*, *saxo2*, *ccdc65*, *nme7*, *ttc26*, *zmynd12*, *ribc1*, *cfap52* (*wdr16*), *rab21* and *dnajc16*] (Patir et al., 2020; van Dam et al., 2019), injected three guides targeting each gene, and found that *ribc1*, *rab21* and *dnajc16* crispants did not have a CDT phenotype, while *ccdc65* was the only crispant that had a severe CDT like the *ift172* mutants (Fig. 7F,G; Fig. S12). The other crispants had variable severity and penetrance of CDT phenotypes (Fig. 7F,G; Fig. S12). However, none of the crispants formed cysts by 5 dpf. To determine whether the variable severity or penetrance is due to guide effectiveness, we evaluated the efficacy of each guide by high-resolution melting curve analysis (HRM) (Fig. 7G; Fig. S12) (Thomas et al., 2014). We observed that 34 of the 42 (80%) guides had strong effectiveness at generating mutations, while 9% did not result in any alterations. The effectiveness of the guide did not correlate with phenotypic severity or penetrance, suggesting that the variability is likely to be associated with gene importance or percentage chimerism of the individuals (Fig. 7G). We also tested guides targeting *tctn1* because we were surprised by the lack of a CDT phenotype in zygotic mutants. Again, loss of *tctn1* did not result in a CDT or cystic phenotype in the crispants (Fig. 7G). To validate the crispant phenotype and address the impact of chimerism on the variable phenotypes, we bred adult *ccdc65* G0s (G0×G0) and scored the frequency and severity of the CDT phenotype in the progeny. From four independent *ccdc65* G0 crosses, we only observed the severe CDT phenotype (Fig. 7H,I). Sequencing of four severe CDT embryos revealed that they all had compound heterozygous frameshifts, resulting in a null allele (Fig. 7I). We also analyzed four normal embryos, which were heterozygous for a frameshift mutation. These data indicate that the variable phenotype severity in *ccdc65* crispants is due to chimerism of the embryos. Consistent with the crispant data, we did not observe pronephric cysts in the F1s. Interestingly, the percentage of CDT phenotype was higher than 25% (expected for a heterozygous in-cross), suggesting that some germ cells in the G0 were homozygous (or compound heterozygous) null. To further confirm that the crispant mutant phenotype is consistent with a zygotic phenotype, we crossed adult *ttc26* G0s. From five independent G0 crosses, we observed a moderate CDT phenotype (Fig. 7H,J). Again, the frequency was often above 25%, suggesting that the germline in these G0s had compound heterozygous-containing cells. In the four CDT mutants analyzed, all had compound frameshift mutations (Fig. 7J), while four normal-looking embryos analyzed were all heterozygous for a frameshift mutation. Together, these data indicate that a G0 crispant screen is an effective way of identifying potential cilia genes.

## DISCUSSION

### Evolutionary importance of TZ genes

**Fig. 8.**
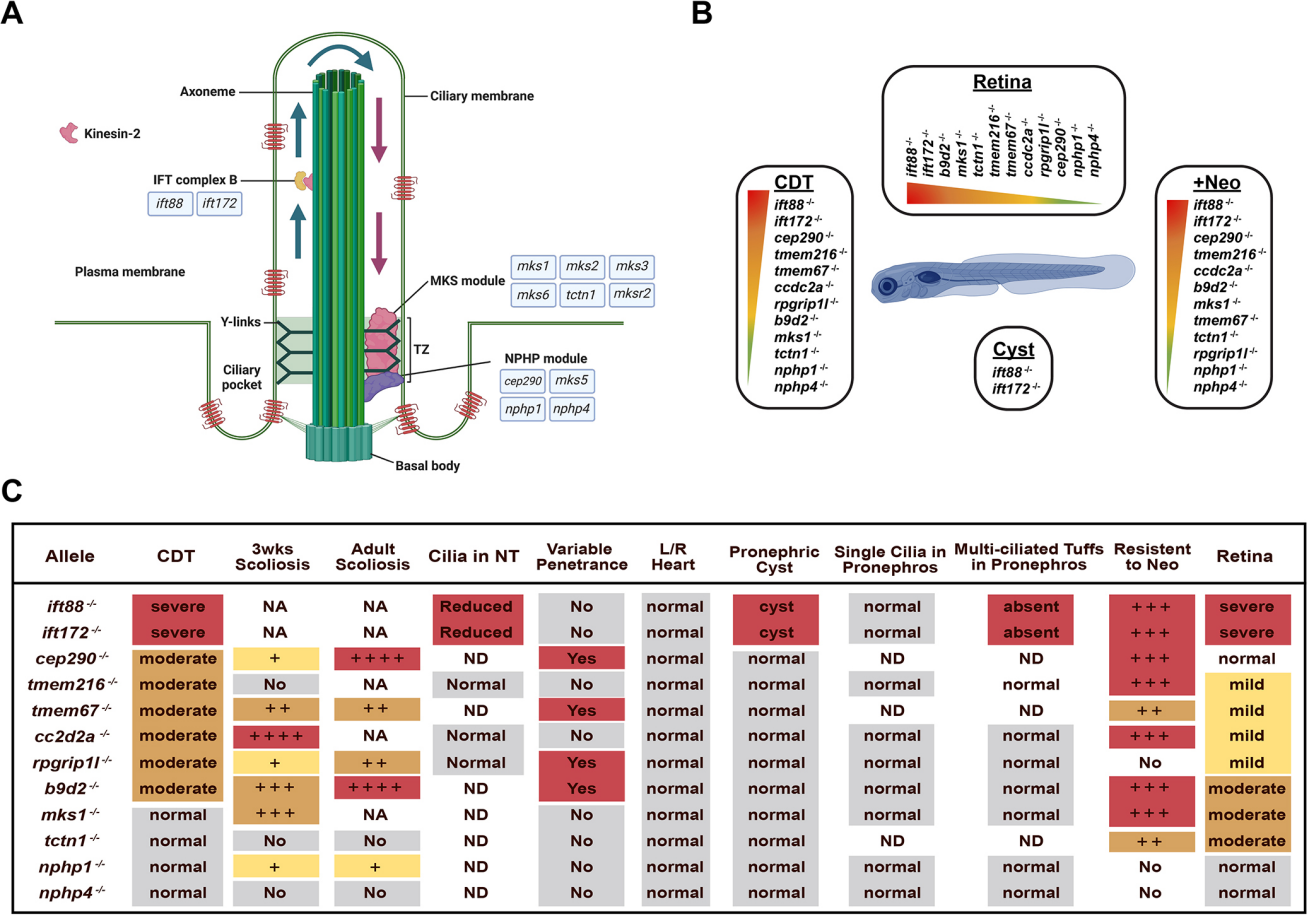
**Summary of data indicating differential requirement for TZ genes.** (A) Illustration of cilium, TZ and genetic components examined in this study. (B) Differential requirement for different TZ genes in different tissues based on phenotypic severity. (C) Table summarizing the variable phenotypes shown in each mutant: red, severe; orange, moderate; yellow, mild; gray, normal looking. Graphic image created with BioRender.com.

Based largely on a combination of *C. elegans* and proteomic analysis, at least three main TZ functional modules have been defined (Fig. 8A): the NPHP module, the MKS module and the CEP290 module.

#### The NPHP module

Humans with recessive mutations in NPHP1 and NPHP4 develop NPHP, and some patients develop retinal degeneration (Otto et al., 2002; Mollet et al., 2002). In *C. elegans*, the NPHP module is redundant with the MKS module, such that there are mild ciliary phenotypes associated with loss of one module, but loss of both results in cilia loss (Williams et al., 2008; Yee et al., 2015). Mouse knockout models of Nphp1 or Nphp4 are viable with no renal phenotypes (Jiang et al., 2008; Won et al., 2011). However, males are sterile, and undergo late-onset retinal degeneration by 8 months of age, with the earliest histological signs at postnatal day (P)21 (Jiang et al., 2008; Won et al., 2011). Our zebrafish data indicate that, like in human, mouse and *C. elegans*, the NPHP module is not essential for viability. However, unlike in mouse, in the zebrafish model, it is also not essential for maintenance of photoreceptors or sperm development. *nphp1*, but not *nphp4*, is involved in low-penetrant scoliosis in zebrafish. Furthermore, we have demonstrated that the lack of phenotypes is not due to redundancy between *nphp1* and *nphp4*, in that double knockouts are viable and fertile. Lack of a phenotype makes the zebrafish NPHP module more similar to the *C. elegans* NPHP module. Note that the viability of our *nphp1* and *nphp4* zygotic null does not match the gastrulation defects published with *nphp1* or *nphp4* morpholinos (Lindstrand et al., 2014; Burcklé et al., 2011); this suggests that the morpholino data contain off-target effects (Robu et al., 2007; Kok et al., 2015) or that the genetic null induces a compensation effect (Rossi et al., 2015).

#### The MKS module

The majority of the MKS module genes were identified in human MKS patients; however, mutations in these genes have also been identified in less severe and phenotypically unique JBTS, BBS and NPHP patients, indicating that there is strong variability in genotype–phenotype correlation. As mentioned above, in *C. elegans*, loss of components of the MKS module alone does not result in ciliary phenotypes; however, loss in conjunction with loss of the NPHP module results in cilia deficiencies. Using a combination of genetic and fluorescent protein-tagged reporter analysis, a hierarchical importance of molecules in the MKS model has been established in *C. elegans*. In brief, MKS-5/RPGRIP1L is the pinnacle molecule in that loss of it results in mislocalization of all other MKS genes tested as well as partial loss of NPHP-4 and NPHP-1. With respect to the order of assembly, below MKS-5, is MKSR-2/B9D2, which influences MKS-2/TMEM216 and MKS-6/CC2D2A (co-dependent on each other) but does not overtly affect NPHP-4. MKS-2 is required for MKS-1 and MKS-3/TMEM67 localization independent of each other (Jensen et al., 2015; Huang et al., 2011; Williams et al., 2011). Although there is a molecular hierarchy of MKS proteins, it is important to mention that phenotypical loss of any of the MKS gene products, in conjunction with loss of a NPHP module, results in a similarly dysfunctional cilium. Further, this is the molecular hierarchy for the amphid and phasmid neurons in *C. elegans*; the molecular hierarchy in other cell types may be different. Recent immunofluorescence studies in different mouse cell types have confirmed that the hierarchy is different in varying cell types with respect to Rpgril1l loss (Jensen et al., 2015). Data in mouse mutants also support these tissue/cell type-specific functions. Mouse knockouts of the MKS module have common phenotypes, as well as unique phenotypes. Mks6/Cc2d2a and Tctn1 are embryonic lethal, and have similar severity of, and timing of, phenotypes such as microphthalmia (E14.5), HH signaling defects such as exencephaly (E11.5) and polydactyly (E14.5), and LR asymmetry defects (Garcia-Gonzalo et al., 2011; Lewis et al., 2019). A variable curved body axis (E10.5), potentially resembling the zebrafish scoliosis, was described in Mks6 nulls, but undescribed in other MKS mutants. Although Mks1, Mks3/Tmem67 and Mks5/Rgrip1l nulls survive to birth, they die shortly after (Garcia-Gonzalo et al., 2011; Weatherbee et al., 2009; Vierkotten et al., 2007). Mks1 and Mks3 nulls have kidney cysts as early as E18.5. The Mks5 null was not analyzed for kidney cysts. Mks1 and Mks5 nulls have variable penetrance cleft palate defects, as well as LR symmetry defects and polydactyly, but not Mks3 nulls. Mks2 and Mksr2 congenital nulls have yet to be described. Together, based on the timing of mortality/severity of the phenotype, this suggests a mouse hierarchy, with Tctn1 and Mks6 being most essential, and Mks1, Tmem67 and Rpgrip1l being less essential, which is almost opposite of the *C. elegans* hierarchy. This could reflect the hierarchy differences in the viability-related tissues. The hierarchy of *rpgrip1l* may also be different due to evolutionary gene differences too. Although *rpgrip1l* is in *C. elegans* (*mks-5*), the homolog *rpgrip1* is a vertebrate-specific gene, which has overlapping function with *rpgrip1l* in some tissues (Wiegering et al., 2018). Further, in the Mks1 mouse knockout study, it was demonstrated that the timing and severity was influenced by strain, raising questioning as to whether the phenotypic differences could be due to modifier genes to a particular MKS in these studies. Our zebrafish data would suggest, based on CDT phenotypic severity and timing, a hierarchy more similar to *C. elegans*, with *tmem217*, *tmem67*, *cc2d2a*, *rpgrip1l* and *b9d2* being most important in complex assembly, and *mks1* and *tctn1* being less important (Fig. 8B,C). However, considering the penetrance contribution, *tmem216* and *cc2d2a* are most important in complex assembly, with *tmem67*, *rpgrip1l* and *b9d2* next, which is more similar to the data from the mouse. We also describe unique phenotypes to certain mutants, such as the small size in the *tmem216* mutants at 3 weeks, which suggests that the hierarchy may be dependent on the tissue or cell type. Among the viable mutants, *b9d2* has the most severe scoliosis, while *tmem67* and *rpgrip1l* are milder, suggesting that *b9d2* is more important in complex assembly than *tmem67* and *rpgrip1l* (Fig. 8C). Based on hair cell sensitivity to neomycin, *tmem216* would be on top, with *cc2d2a*, *b9d2* and *mks1* to follow, then *tmem67* and *tctn1*, and *rpgrip1l* below (Fig. 8B,C). Interestingly, the cones of the retina have almost opposite hierarchy, with *b9d2*, *mks1* and *tctn1* at the top, and *tmem216*, *tmeme67*, *cc2d2a* and *rpgrip1l* below (Fig. 8B,C). Most important is that these data make it clear that different MKS proteins contribute differently to the degree or tissue-specific phenotypes, implying either redundancy or unique gating capabilities of individual MKS molecules. Most surprising from our data was that although *Tctn1* is essential in mouse, *tctn1* is dispensable in zebrafish. Potentially, other TCTN proteins play a more essential role or provide compensation in zebrafish. Mutants with loss of *mks1* and *tctn1* have phenotypes in primary cilia-related cells (retina and hair cells), but lack phenotypes in motile cilia, suggesting that *mks1* and *tctn1* have more important roles in primary cilia than in motile cilia. This is more consistent with mouse and *C. elegans* phenotypes.

#### The CEP290 module

Mutations in CEP290 in humans have been identified in a number of ciliopathies with different phenotypes, including MKS4, JBTS5, LCA10 and SLSN6 (Rachel et al., 2012). Interestingly, there is strong phenotypic variation between these patients that cannot be explained by the location or type of mutation in CEP290. Some of the phenotypes of these patients include kidney cysts, retinal degeneration and spinal deformities. In *C. elegans*, CEP-290 functions as a component of the MKS module, in which cilia loss is only observed when the NPHP module is also lost and in the hierarchy functions below MKS-5 but above MKS-2 (Li et al., 2016). However, proteomic analysis of human cells indicates that the CEP290 interactome is independent of the MKS model (Sang et al., 2011). Surprisingly, all three mouse knockouts of Cep290 are viable as homozygotes, with a percentage dying due to hydrocephalus after birth. Those that survive have a normal lifespan, but have retinal degeneration and late-onset cystic kidneys (Rachel et al., 2015; Lancaster et al., 2011; Sang et al., 2011; Chang et al., 2006). The zebrafish *cep290* mutant has a partial penetrance phenotype conceptually like the mouse's partial penetrant hydrocephalus-derived lethality and the variability in human patient phenotypes. Further, we provide data to suggest that this penetrance phenotype is influenced by genetic modifiers. Therefore, this zebrafish *cep290* mutant is a good model to understand the partial penetrance and variable phenotypes observed between human individuals. Our data also indicate differential requirement for *cep290* in different cell types (Fig. 8C). For example, *cep290* is important in neomycin resistance and CDT, but dispensable for maintenance of the embryonic retina (Fig. 8B,C). Consistent with the mouse, some *cep290* null zebrafish are viable and display adult retinal degeneration (Lessieur et al., 2019). Our *cep290* zygotic null does not match published phenotypes of *cep290* morpholino data from different laboratories (Baye et al., 2011; Cheng et al., 2012; Schäafer et al., 2008; Sayer et al., 2006), again suggesting either morpholino off-target effects or compensation for zygotic loss.

### Zebrafish phenotypes

#### Curved body phenotype

We observed a gradient of timing and severity of body curvature, between embryonic, prior to bone formation, to adulthood scoliotic-like spine curvature in the different TZ mutants (Fig. 8B,C), suggesting that different TZ proteins play more essential roles in the TZ regarding the body curvature phenotype. It is unclear whether these embryonic versus adult phenotypes are directly related, but recent studies have shown that *scospondin* (*sspo*) nulls have a curved down embryonic phenotype, while *scospondin* missense mutants form a juvenile scoliotic phenotype, suggesting that they are a continuum of the same defect (Cantaut-Belarif et al., 2018; Troutwine et al., 2020; Rose et al., 2020). Further, these studies demonstrate that *scospondin*, which is secreted out of a group of cells (sub-commissural organ) in the 3rd and 4th ventricle, is important for the formation of Reisner fibers along the entire length of the neural tube and contributes to proper axis formation. Immotile cilia in the neural tube can result in a curved body axis/scoliosis, potentially due to lack of Reisner fiber transport (Lu et al., 2020; Grimes et al., 2016). However, we did not observe a cilia motility defect in *cc2d2a* mutants, suggesting that a defect in signaling or transport through the cilia may also contribute to curved body axis. Mutants in *urotensin* and/or *stat3* also have curved down body or scoliosis, indicating the importance of these signaling molecules in axis formation (Lu et al., 2020; Liu et al., 2017). Interestingly, we observed upregulated expression of *urp2* in *ift88*, *ift172*, *b9d2*, *tmem216*, *rpgrip1l* and *cc2d2a* mutants at 5 dpf, suggesting a role of the urotensin pathway in the CDT phenotype downstream of the ciliary defect. Further RNA-seq analysis of the 2 dpf *rpgrip1l* group also defined 16 genes in the 2 dpf CDT cluster that shared the binding motif of transcription factor Pax2, which is activated by Wnt signaling (Cai et al., 2002). Deciphering whether these signaling pathways are defective in TZ mutants is an important next step. Towards this, the multi-guide injection will provide a fast approach to decipher the upstream and downstream components of signaling pathways involved. Interestingly, the variability in the timing of post-embryonic scoliotic phenotypes suggests that scoliosis is not associated with a specific developmental stage, but maintenance of spine curvature is a process throughout life. The fact that IFT and TZ mutants have a clear difference in phenotype severity (severe CDT and moderate or mild CDT, respectively) suggests that they play a different role in this process. The TZ genes may play a more important role in molecular gating of important signaling molecules through the TZ, while IFT complexes affect transport through and formation of cilia.

#### Retinal degeneration

Interestingly, retinal degeneration is present in most ciliopathy disorders, indicating that photoreceptor cells are highly sensitive to ciliary dysfunction. Zebrafish have the ability to regenerate photoreceptors; therefore, regeneration can overcome mild retinal degeneration. This could be why a retinal degeneration phenotype is not observed in *nphp1* or *nphp4* mutants. Alternatively, there could be some form of genetic compensation similar to *C. elegans*, in which loss of NPHP1 or NPHP4 requires the additional loss of the MKS module in order to observe a phenotype (Williams et al., 2011; Sang et al., 2011; Szymanska et al., 2014; Reiter et al., 2012; Huang et al., 2011). Interestingly, loss of any of the MKS module proteins analyzed in this study resulted in some degree of cone defects, while only loss of *mks1*, *b9d2*, *tmem216* and *cc2d2a* resulted in rod defects. This suggests that the TZ proteins may have varying degrees of importance in generating the TZ gating complex in different retinal cell types, or there are different levels of redundancies in rods versus cones. The *mks1* mutant is interesting in that there is no CDT phenotype at 5 dpf; however, a clear cone phenotype exists at 5 dpf. This suggests that the importance of *mks1* in gating is different in CDT-determining tissues (potentially neural tube) versus the retina. Alternatively, because *mks1* has a strong role in neuromast sensitivity to neomycin and retinal degeneration, its function could be restricted to non-motile cilia. This observation extends to a lesser extent into *tctn1*, which has mild neuromast resistance and mild retinal degeneration (Sang et al., 2011), but no CDT phenotype. Our transcriptional analysis defined different gene clusters that are associated with disease severity. In unrelated studies, this retinal signature has been useful in identifying a mild retinal degeneration in mutants that display no overt morphology defect. Unexpectedly, the signature did not correlate with loss of a specific rod or cone subtype. This could suggest differential transcriptional sensitive networks, or that degeneration of one cell type also creates disrupted transcriptional signatures in surrounding cell types.

#### Pronephric cysts

Polycystic kidney disease (PKD) has been one of the hallmark diseases within ciliopathies. Interestingly, within zebrafish IFT mutants, we observed pronephric cysts in all embryos, but, in the ten TZ mutants, we never observed a cyst. Correlative with this, we observed a lack of anterior multi-ciliated tufts in IFT mutants but not in TZ mutants (Fig. 8C). Lack of these MCCs has previously been implied as a contributor to pronephric cyst formation (Liu et al., 2007). Importantly, reestablishment of the MCC fate through manipulation of Jagged2/Notch signaling was able to rescue cyst formation in a double mutant (Liu et al., 2007). This suggests that IFT-dependent formation of the cilia or signaling through cilia is important for MCC fate establishment or maintenance. Why IFT mutants, but not TZ mutants, have loss of MCC is unclear, but potentially this is associated with the need for IFT proteins to form the multiple cilia on MCCs, whereas TZ proteins may not be necessary for building the structure but play a role in gating signaling molecules through the cilia. The cilia-dependent cell fate determination may explain the observation in the mouse that loss of Ift88 prior to P14 results in rapid and widespread cyst formation, whereas loss of Ift88 after P14 results in late-onset and focal cyst formation (Davenport et al., 2007; Sharma et al., 2013). Therefore, prior to P14, formation of a cilia is important for non-cystic fate establishment, whereas after P14 the non-cystic fate is established, and cilia loss only comes into play after injury and dedifferentiation to repair. The zebrafish IFT mutants would be a convenient model to understand the cilia-dependent cell fate establishment.

#### Penetrance

One perplexing aspect of ciliopathies is that mutations in a single gene can be responsible for multiple clearly defined genetic disorders. For example, truncating mutations in CEP290 can cause the very severe perinatal lethal phenotype in MKS4, or a gradient of phenotypic severities associated with LCA10, NPHP6, SLSN6 and JBT5. Genetic modifiers are likely to influence the severity of these patients with CEP290 mutations. Recently a genetic modifier gene [*Barttin* (*Bsnd*)] of mouse Cep290 null phenotype was identified; however, we did not observe a statistically significant change in expression of this gene in the *rpgrip1l* dataset (Ramsbottom et al., 2020). This could reflect different modifiers for *cep290* versus *rpgrip1l*. Further, Cardenas-Rodriguez et al. (2021) recently identified compensation factors (*arl3a*, *arl3b*, *arl13a*, *arl13b* and *unc119b*) for the mild zebrafish *cep290* zygotic mutant phenotypes compared to the more severe morpholino-derived phenotype. However, again we did not observe statistical differences in these genes in our *rpgripl1* DEG list. Together, these results indicate that there are likely to be multiple genetic modifiers or different modifiers for different ciliopathy genes. Towards this, our data suggest that a genetic modifier influences the CDT in *cep290* and *rpgripl1* zebrafish mutants. Importantly, these zebrafish TZ mutants can be used to identify ciliopathy modifier genes. Our transcriptional analysis defined a potential modifier transcriptional signature. In the modified cluster, there was (1) decreased expression of the TZ protein gene *cep76*, (2) decreased cAMP-dependent kinase *prkar1aa* and (3) altered Wnt signaling/Frizzled-related protein gene *sfrp1b*, as well as upregulated expression of *musk*, encoding a Frizzled-like domain protein, all potential modifier networks. Together with the CDT signature, these data indicate that Wnt signaling is important in many of the ciliary phenotypes. Future studies using crispant or mRNA overexpression can be used to identify transcripts responsible for the correction of CDT phenotype.

#### Zygotic mutant versus morpholino-derived mutant versus CRISPR-derived mutant

Except for the curved body plan, almost all the TZ zygotic mutants we generated do not phenocopy morpholino-derived phenotypes (Lindstrand et al., 2014; Burcklé et al., 2011; Sayer et al., 2006; Baye et al., 2011; Cheng et al., 2012; Schäfer et al., 2008). Maternal protein could explain the phenotypic difference due to maternal mRNA/protein rescue in zygotic mutants. However, we bred *rpgrip1l*^−/−^ females to *rpgrip1l*^+/−^ males, to obtain maternal zygotic null embryos. We did not observe LR defects or hydrocephaly in *rpgrip1l*^−/−^ embryos, suggesting that the lack of these phenotypes in zygotic mutants is not due to maternal mRNA/protein, but to *rpgrip1l* loss. We observed the same results in maternal-zygotic nulls for *nphp1*, *nphp4* and *tctn1*, indicating that, at least for these genes, maternal contributions are not rescuing the phenotype. Further, the hydrocephalus and LR axis phenotypes are likely an off-target effect of morpholinos because we observe these phenotypes often with many non-specific morpholinos. However, pronephric cysts are not often observed as a side effect of morpholinos, suggesting that they are specific to the gene targeted. In PKD, there is a third hit model, in which kidney injury or other events are required for a cyst to form. Potentially, morpholinos induce a stress that, in conjunction with targeted gene knockdown, triggers pronephric cysts. As an alternative to morpholinos, we have demonstrated that multiple guide-derived CRISPR F0 crispants recapitulate the zygotic phenotype, including CDT, cysts or scoliosis, without the additional non-specific phenotypes. Further, we have shown that the crispant technique can rapidly validate putative cilia genes. With crispants, we identified that loss of 11 of the 14 putative cilia genes had a clear role in cilia. With the two genes in which we generated zygotic mutants (*ccdc65* and *ttc26*), the variability in CDT severity within a crispant clutch is due to chimerism of biallelic loss of function alleles, because the zygotic mutant produced a consistent phenotype more similar to the most severe phenotype observed in the crispant clutch. The zygotic CDT severity of the *ccdc65* mutant was stronger than that of the *ttc26* mutant, but this was also observed in the crispant clutches. Hence, we could argue that the zygotic phenotype is often the most severe phenotype observed in the crispant clutch.

## MATERIALS AND METHODS

### Zebrafish lines and maintenance

All zebrafish work was performed at the University of Alabama at Birmingham (UAB) in the Zebrafish Research Facility (ZRF). Adult fish and embryos were maintained as described by Westerfield et al. (1995) by the ZRF Animal Resources Program, which maintains full Association for Assessment and Accreditation of Laboratory Animal Care (AAALAC) accreditation and is assured with the Office of Laboratory Animal Welfare (OLAW). All animal studies have UAB Institutional Animal Care and Use Committee (IACUC) approval. All knockout lines were generated on the AB strain. Embryos were yolk injected at the one-cell stage and analyzed between 4 h post fertilization (hpf) and 5 dpf. Adults were analyzed at 3 wpf, 7 wpf and 3 months post fertilization (mpf). All experiments and analyses were performed on embryos or adults from multiple clutches.

### Generation of cilia-related mutant alleles

Gene knockouts were generated as described previously (Thomas et al., 2014). The overall strategy was to design effective guides to a 5′ coding exon to induce frameshift mutations resulting in an early truncated and a non-functional protein. gRNA target sites were identified using the Zhang laboratory gRNA design tool (http://crispr.mit.edu) and the protospacer adjacent motif (PAM) site listed in figures. The CRISPR gRNA sequences were cloned into pDR274 (Addgene #42250) or directly ordered from Integrated DNA Technologies (IDT). The Cas9 mRNA was transcribed from pT3TS-nCas9n (Addgene #46757). After cloning specific target plasmids/guides into pCS2 variant vector, mRNA was generated by *in vitro* transcription off NotI-hf linearized DNA using the Invitrogen mMESSAGE mMACHINE™ SP6 Transcription Kit (Thermo Fisher Scientific, AM1340) and purified with the MEGAclear™ Transcription Clean Up Kit (Thermo Fisher Scientific, AM1908). Approximately 1-2 nl nuclease mRNA (or sgRNA/Cas9 mRNA) was microinjected into the yolk of one-cell-stage zebrafish embryos. For indel efficiency evaluation, genomic DNA was extracted from ∼24 of the 5 dpf-injected embryos and evaluated with HRM (see below). The remaining embryos (F0s) from the clutches were raised. F1 progeny carrying out of frame indel alleles were maintained and propagated. To ‘clean up’ genetic background, all lines were bred at least two generations to the WT strain. In the case of *nphp1* and *nphp4*, in which homozygous out-of-frame mutations did not produce a phenotype, we generated a second null allele in a downstream exon (Fig. S1). In both cases, the second alleles had the same phenotype as the first. We obtained multiple Cas9-derived mutations for each targeting site (Fig. S2); however, we only maintained one out-of-frame allele for each target site per gene as described in Fig. S1.

### Cas9 ribonucleoprotein (RNP) preparation and microinjection for G0 knockout screening

Alt-R crRNA target sites were designed with the IDT Alt-R CRISPR HDR Design Tool (https://www.idtdna.com/pages/tools/alt-r-crispr-hdr-design-tool). Alt-R CRISPR-Cas9 crRNA, tracrRNA (IDT, 1072532) and Alt-R S.p. Cas9 Nuclease V3 (IDT, 1081058) were prepared following the manufacturer's instructions. Then, 3 µM gRNA was obtained by diluting 100 µM crRNA and 100 µM tracrRNA in Nuclease-Free Duplex Buffer (IDT, 11-05-01-03), heating at 98°C for 5 min, then cooling to room temperature. The final sgRNA concentration remained the same when one or three guides were used. Then, 0.5 µl Cas9 protein was diluted with Cas9 working buffer (20 mM HEPES; 150 mM KCl, pH 7.5) to yield a working concentration of 0.5 µg/µl. The diluted Cas9 protein working solution was mixed 1:1 with 3 µM gRNA solution and then incubated at 37°C for 10 min. Microinjection was performed by injecting ∼1 nl of RNP complex into the yolk of one-cell-stage embryos. The RNP complex was freshly prepared and left on ice until microinjection (Wu et al., 2018). IDT crRNAs used for multiple-guide RNA injections are listed in Table S3. We selected the 14 putative cilia genes based on the following selection criteria, excluding (1) genes in which mouse knockouts indicated ciliary or non-ciliary function; and (2) genes in which zebrafish cilia mutants have been described. Therefore, we selected six putative genes (*ankfn1*, *cfap57*, *fhad1*, *c1orf194*, *pacrg*, *saxo2*) based on genes that co-express with *Foxj1* in multiple mouse cell types (Patir et al., 2020), six genes [*ccdc65*, *nme7*, *ttc26*, *zmynd12*, *ribc1*, *cfap52* (*wdr16*)] that are listed in the CiliaCarta top 50 genes (van Dam et al., 2019) and two genes (*rab21*, *dnajc16*) identified in the INDICiliATOR algorithm (Jeremy Reiter, University of California, San Francisco, personal communication) that is based on proteomic interactions.

### Genotyping with HRM

To isolate genomic DNA from adults, tail clippings from each fish were incubated at 98°C for 20 min in 40 µl of 25 mM NaOH in a 96-well plate, then neutralized with 40 µl of 40 mM Tris-HCl. Early-stage or stained embryos were incubated at 55°C for 2 h in 25 µl ELB (10 mM Tris-HCl pH 8.3, 50 mM KCl, 0.3% Tween 20, 0.3% NP40, 1 mg/ml proteinase K) in 96-well plates, then incubated at 95°C for 15 min to inactivate the proteinase K. PCR reactions contained 1 μl LC Green Plus Melting Dye (Biofire Defense, BCHM-ASY-0005), 1 µl of 10× enzyme buffer, 0.2 µl dNTP mixture (10 mM each), 0.3 µl MgCl_2_, 0.3 µl of each primer (10 µM), 1 µl genomic DNA, 0.05 µl Genscript Taq (E00101) and water up to 10 µl. The PCR reaction protocol was 98°C for 30 s, then 45 cycles of 98°C for 10 s, 59°C for 20 s and 72°C for 15 s, followed by 95°C for 30 s and then rapid cooling to 4°C. Following PCR, melting curves were generated and analyzed using a LightScanner instrument (Idaho Technology) over a 65-95°C range. Primers used for identifying zebrafish knockout lines were designed with Primer3 and are listed in Table S1.

### Identification of alleles, NMD and maternal RNA contribution

To identify mutated alleles, DNA was extracted from a small piece of tail from F1 or F2 progenies in each mutant with proteinase K (details above). The DNA fragment flanking the targeting site was PCR amplified using Ex Taq DNA Polymerase (Takara Bio, RR001A), purified with Promega Wizard SV Gel and PCR Cleanup System (Promega, A9282), and examined on a 1% agarose gel (for examining alternative splicing) and sequenced by the UAB Heflin Center for Genomic Sciences Sanger Sequencing Core. For analysis of maternal RNA contribution, RNA from homozygous mutants at 2 dpf and 5 dpf was harvested. To determine whether the mutated allele is undergoing NMD, a small piece of tail was cut from a single heterozygous fish (of each allele). RNA was extracted from each tail using Trizol Reagent (Life Technologies, 15596026), and cDNA was synthesized from each RNA sample using a High-Capacity cDNA Reverse Transcription Kit (Life Technologies, 4368814). For PCR, 1 μl cDNA from each sample was amplified with HS Phusion™ High-Fidelity DNA polymerase (Thermo Fisher Scientific, F530S) along with the primers listed in Table S2. PCR cycling conditions were 98°C for 30 s, followed by 25 cycles of 98°C for 10 s, 60°C for 30 s, 72°C for 15 s and then 72°C for 10 min. The products of all first-round PCR reactions were then gel purified. We used 25 ng of the purified PCR products for sample indexing in preparation for NGS using Illumina TruSeq indexing primers. PCR cycling conditions were 98°C for 30 s, then 8 cycles of 98°C for 10 s, 60°C for 30 s and 72°C for 15 s, followed by 72°C for 10 min. The final PCR products were gel purified and run on the Illumina MiSeq platform using 2×150 bp paired end reads. For each mutant sample, the percentage of NGS reads containing WT or mutant sequences was analyzed using Cas-Analyze (http://www.rgenome.net/cas-analyzer/#!; Park et al., 2017). In conditions of no NMD, we expect a heterozygote to have a 1:1 ratio of mutant to WT alleles. Ideally, complete NMD of mutant alleles would have a ratio of 1:high. We categorized mutants as no NMD (ratio 1:≤1.5), partial NMD (ratio 1:>1.5 but<3) or strong NMD (∼1:>5). We also confirmed that the chromogram of Sanger sequencing could accurately predict NMD as an alternative to this NGS approach (Fig. S3C). Primers used for NGS and Sanger sequencing were designed with Primer3 and are listed in Table S2.

### RNA whole-mount *in situ* hybridization

Digoxigenin-labeled RNA probes were used in RNA *in situ* hybridization of standard methods on appropriately aged embryos as described (Thisse and Thisse, 2008). cDNA was amplified from a zebrafish WT cDNA library and cloned into Zero Blunt TOPO^®^ vector (Invitrogen, 450245). For the sense probe, the plasmid was linearized with NotI-HF and transcribed with SP6 enzyme. For the antisense probe, the plasmid was linearized with SpeI and transcribed with T7 enzyme. The *cmlc2* plasmid (a gift from Dr Dan Gorelick, Baylor College of Medicine, Houston, TX, USA) was used for the synthesis of genomic cDNA to compound antisense and sense constructs of RNA probe. Brightfield images of the embryos were obtained using a Nikon SMZ-18 Zoom Stereo Microscope.

### Neuromast staining

One milliliter of neomycin [10 mM stock in dimethyl sulfoxide (DMSO); Thermo Fisher Scientific, BP2669-5] was added to 49 ml sterile E3 egg water to prepare a working solution with a final concentration of 200 µM. Approximately 100 embryos [homozygous mutant and sibling controls (includes +/− and +/+)] at 5 dpf were placed in a 60×15 mm Petri dish. For neuromast staining, embryos were left for 1 h at 28.5°C, followed by three washes and allowed to recover in sterile E3 egg water for 1 h. Embryos with or without neomycin were incubated in E3 blue containing 50 μg/ml 2-[4-(dimethylamino) styryl]-N-ethylpyridinium iodide (DASPEI; Sigma-Aldrich, D0815; specifically to label hair cells) for 30 min, followed by two to three washes. Embryos were anesthetized in 0.4% tricaine (3-aminobenzoic acid ethyl ester; Sigma-Aldrich) and immediately scored by counting the number of visible neuromasts out of total neuromasts on the head and trunk regions of embryos using a Nikon SMZ-18 Zoom Stereo Microscope with GFP filter (further imaging details below).

### AR staining

We performed AR staining as published previously (Sakata-Haga et al., 2018). Adult zebrafish were euthanized and placed in fixative solution (5% formalin, 5% Triton X-100 and 1% KOH), and were slowly rocked at 42°C for 2-3 weeks. Fixing medium was changed every 3-5 days. Once samples were sufficiently bleached, they were descaled and washed with deionized water and placed in B staining medium (20% ethylene glycol and 1% KOH; without AR) and rocked at room temperature for 15 min. The samples were then transferred to B staining solution (20% ethylene glycol, 1% KOH and 0.05% AR) and rocked at room temperature for 15 min. Samples were then washed one or two times with clearing solution (20% Tween 20 and 1%KOH) and rocked in clearing solution for 4-8 h until excess dye was removed at 42°C. Samples were imaged at 0.75× magnification with the TRITC filter using a Nikon SMZ-18 Zoom Stereo Microscope (further imaging details below). Images were stitched together pairwise using the ImageJ Stitching plugin (Preibisch et al., 2009).

### μCT scanning

Adult zebrafish were euthanized at 3 mpf and fixed with 1× Formal-Fixx solution (Epredia, 9990918) at 4°C overnight followed by rinsing twice with PBS for 10 min. All samples were scanned by a Scanco µCT40 desktop cone-beam μCT scanner (Scanco Medical, Brüttisellen, Switzerland; using µCT v5.44) at the UAB Small Animal Phenotyping Core. Scans were automatically reconstructed into 2D slices, and all slices were analyzed using the µCT Evaluation Program (v.6.5-2; Scanco Medical). The fish were placed in a 30 mm-diameter scanning holder and scanned at the following settings: 15 µm voxel size, 70 kVp, 114 µA, 1000 projections/180° with an integration time of 200 ms. The region of interest was outlined to include all parts of the fish. Bone was thresholded at 160.97 mgHA/cm^3^, and the 3D analysis was performed to obtain bone volume and density. 3D images were obtained from RadiAnt Dicom Viewer.

### Whole-embryo immunohistochemistry staining

Zebrafish embryos to analyze cilia in the pronephric duct, otolith, neural tube and hair cells were fixed in 4% paraformaldehyde (PFA) overnight at 4°C, followed by stepwise dehydration into methanol for storage in −20°C. Embryos underwent stepwise rehydration into PBS and were washed in PBST (PBS + 0.1% Tween 20) 4× for 5 min each. After 2 h blocking with 1% DMSO, 10% sheep serum in PBS, embryos were incubated with anti-acetylated alpha-Tubulin antibody (1:500; Sigma-Aldrich, T-6793) overnight at 4°C, and then incubated with fluorophore-conjugated secondary antibody (1:500; Molecular Probes) overnight at 4°C. Subsequently, embryos were washed and stained with 4, 5-diamidino-2-phenylendole (DAPI) for 10 min (if nucleus measurement necessary) prior to imaging.

### Embryo and adult zebrafish retina staining

Immunohistochemistry and staining were performed as before (Song et al., 2020). Larvae were fixed in 4% PFA and equilibrated in PBS+30% sucrose for 2 h. After washing with PBS, larvae were embedded in Tissue Freezing Medium (Electron Microscopy Sciences, Hatfield, PA, USA). Adult eyes were fixed for 2 h in 4% PFA. Fixation protocols varied depending on the primary antibodies being used. For Zpr1 and Zpr3, samples were fixed in 4% PFA in 0.8× PBS at 4°C overnight. For PNA, embryos were fixed in 4% PFA in 0.8× PBS at 4°C for a maximum of 2 h. Eyes were washed in PBS and equilibrated in 5% sucrose/PBS for 3 h at room temperature and then in 30% sucrose overnight at 4°C. Eyes were washed in a 1:1 solution of 30% sucrose:Tissue Freezing Medium overnight at 4°C and subsequently embedded for cryosectioning. Cryosections (10 μm) were cut and dried at room temperature overnight. Slides were washed 3×10 min in PBS and then incubated in blocking solution (2% bovine serum albumin, 5% normal goat serum, 0.1% Tween 20 and 0.1% DMSO in PBS) for 1 h. The following primary antibodies were used: mouse monoclonal anti-Zpr1 [1:100; Zebrafish International Resource Center (ZIRC), Eugene, OR, USA], mouse monoclonal anti-Zpr3 (1:100; ZIRC), mouse monoclonal anti-4C4 (1:1000; a gift from Dr Peter Hitchcock, University of Michigan, Ann Arbor, MI, USA), rabbit polyclonal anti-L-plastin (1:1000; GeneTex, Irvine, CA, United States, GTX124420), mouse monoclonal anti-Pcna (1:100; Sigma-Aldrich, clone PC-10) and PNA-lectin conjugated to Alexa Fluor 568 (1:100; Thermo Fisher Scientific). Alexa Fluor-conjugated secondary antibodies were used at 1:500 in blocking buffer and incubated for at least 1 h. Slides were counterstained with DAPI to stain nuclei. Sections were imaged on a Zeiss Imager Z.2 equipped with an Apotome using Zen2 software and post-processed in ImageJ. All analysis was performed only on sections that included or were immediately adjacent to the optic nerve.

### Light, immunofluorescence and confocal imaging

For gross imaging of 2 dpf embryos, embryos were dechorionated at described stages with incubation in 0.03% pronase (Sigma-Aldrich, P5147) for 6 min at 1 dpf. Embryos and adults were anesthetized using 0.4% tricaine. For light and immunofluorescence imaging, embryos were mounted in 1% low-melting-point agarose in a 60×15 mm Petri dish. Gross images and images with DASPEI or AR staining were taken on a Nikon SMZ-18 Zoom Stereo Microscope. For confocal imaging, embryos were mounted on a glass-coverslip-bottomed dish and imaged using a Nikon A1 confocal with a 60× oil-immersion objective. For quantification, all images were acquired at the same magnification, laser power, exposure time and gain. After each embryo was imaged, embryos were removed from the agarose to generate genomic DNA for genotyping. Further figure processing and analysis was performed using Nikon NIS Element and ImageJ.

### Imaging cilia motility

Live 2 dpf WT sibling and mutant embryos were immobilized in 1% low-melting-point agarose for imaging. Differential interference contrast images of motile cilia in the posterior neural tube were captured at 60 frames/s using a Zeiss Axiocam HSm high-speed camera on a Zeiss Imager M1 AX10 microscope with a 63× water-dipping objective.

### Bulk RNA-seq and analysis

RNA samples were prepared with a Qiagen RNeasy Plus Mini Kit. The RNA library was prepared with Illumina RNA with PolyA selection package and sequenced with Illumina HiSeq 2×150 bp, single index at Genewiz. Raw sequencing reads were trimmed TrimGalore 0.4.4, aligned with Star 2.7.3a to *Danio rerio* GRCz11 and calculated with HTSeq-count 0.12.3 with default setting. The count was used to obtain DEGs with DESeq2 in R. GO analysis was performed with DAVID Bioinformatics Resources 6.8. Gene expression was quantified via RSEM v1.3.2 (a tool for quantifying transcript abundances from RNA-seq data) (Li et al., 2011) with the default setting and the normalized count was used to obtain heatmap with pheatmap in R.

### Fluorescence quantification and statistical analysis

GraphPad Prism 9 was used for the generation of all graphs and statistical tests. Numbers and statistical tests are indicated in figure legends. All experiments involving phenotyping of mutants were first scored blind to genotype, then the embryos were sacrificed for genotyping.

### Western blotting analysis

HEK293 cells and 2 dpf zebrafish embryos were homogenized in RIPA buffer (Cell Signaling Technology, 9806) containing Protease Inhibitor Cocktail (Roche, 04693159001) followed by short sonication. Sample supernatants were collected after centrifugation at 18,400 ***g*** for 20 min. Protein (30 μg) was separated on a NuPAGE™ Mini Protein Gel (Invitrogen, NP0322BOX) before being transferred to PVDF membrane (Millipore, IPVH00010). Membranes were blocked in TBS containing 5% non-fat milk powder (Lab Scientific) for 1 h. Immunoblotting was performed at 4°C overnight using antibodies against TMEM67 (1:1000; 13975-1-AP), TCTN1 (1:1000; 15004-1-AP), B9D2 (1:1000; 22508-1-AP) and MKS5/RPGRIP1L (1:1000; 29778-1-AP) from Proteintech; NPHP1 (1:1000; GTX65891) from GeneTex; IFT172 (1:1000; bs-15560R) from Thermo Fisher Scientific; NPHP4 (1:1000; H00261734-M01A) from Abnova; CEP290 (1:1000; ab84870) from Abcam; and GAPDH (1:2000; 2118) from Cell Signaling Technology. Antibody against Ift88 (1:3000) was generously gifted by Dr Brian Perkins (Cole Eye Institute, Cleveland Clinic, Cleveland, OH, USA). The membranes were incubated with a secondary antibody at room temperature for 1 h after washing with TBS containing 0.5% Tween 20. Protein bands were visualized using Pierce Clarity Western ECL substrate (Bio-Rad, 170-5061).

## Supplementary Material

10.1242/dmm.049568_sup1 Supplementary information
