# Supplementary material for: Variable phenotypes and penetrance between and within different zebrafish ciliary transition zone mutants
Source: Dis Model Mech. 2022 Dec 19;15(12):dmm049568. doi: 10.1242/dmm.049568 (PMC9844136; doi:10.1242/dmm.049568)
Supplement: Supplementary information [file dmm-15-049568-s1.pdf]

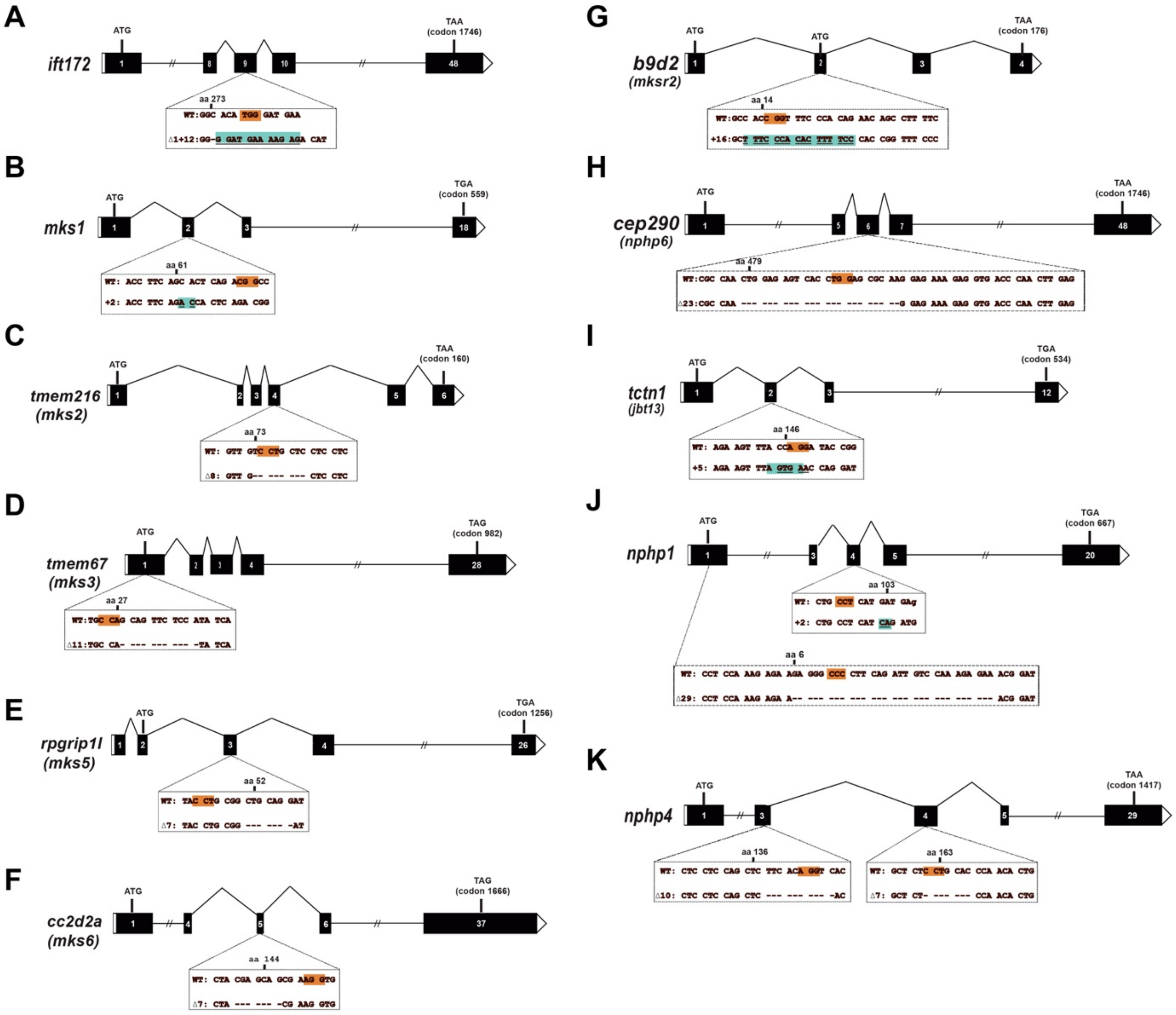

Fig. S1. Generation of 11 cilia-related mutants in zebrafish. Schematic diagram of the target site in zebrafish (A) *ift172*, (B) *mks1*, (C) *mks2/tmem216*, (D) *mks3/tmem67*, (E) *mks5/rpgrip1l*, (F) *mks6/cc2d2a*, (G) *mksr2/b9d2*, (H) *cep290/nphp6*, (I) *tctn1/jet1*, (J) *nphp1* and (K) *nphp4* loci. The nucleotide sequence of wildtype and mutant alleles at target sites are shown. The orange highlights PAM site, green indicates nucleotides inserted, and dash indicates nucleotides deleted. All mutant alleles are out of frame mutations resulting in the predicted protein truncation, and the frame-shift amino acid site for each allele is labelled on WT sequence.

Disease Models & Mechanisms • Supplementary information

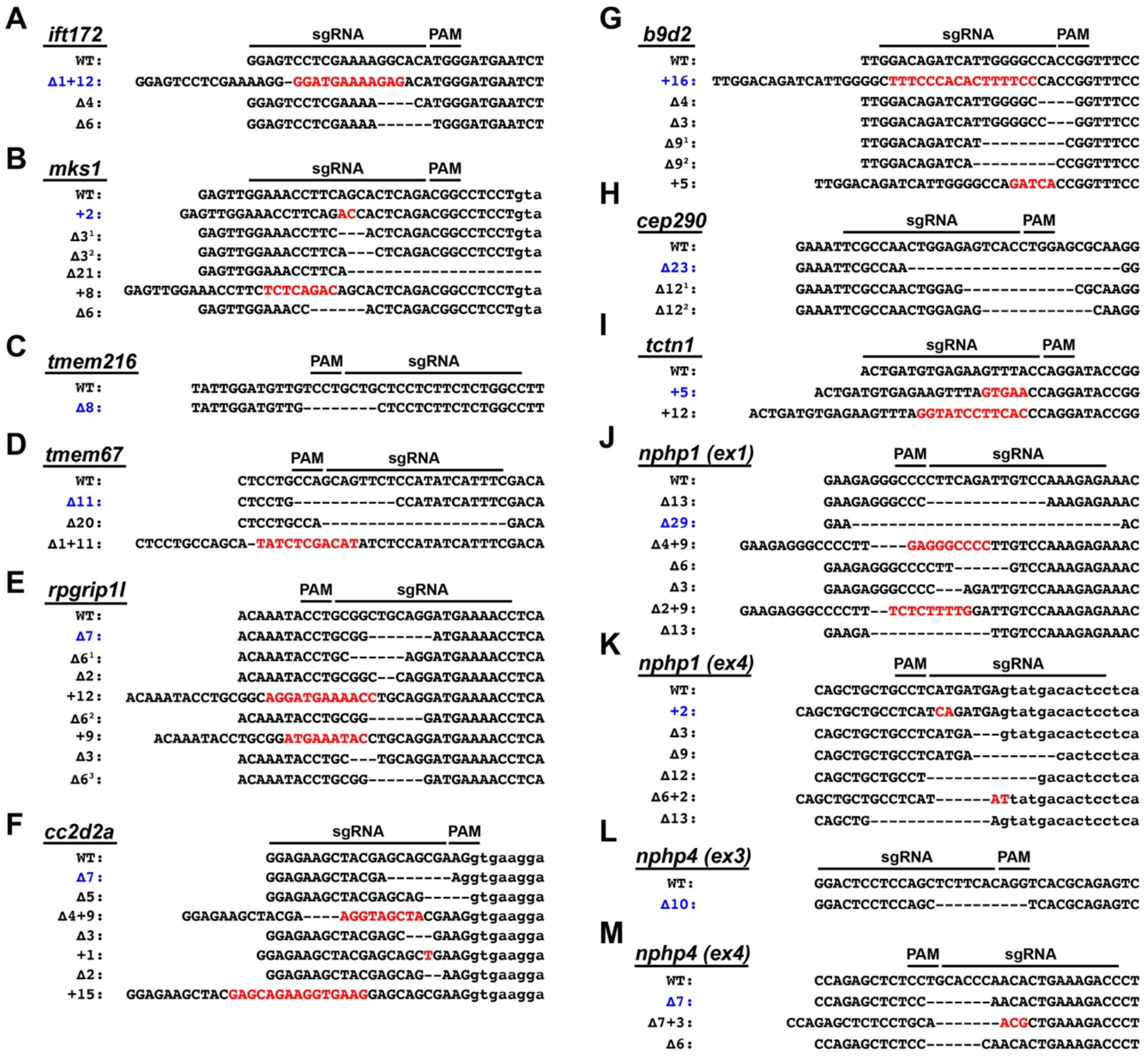

Fig. S2. Alignment of wild-type and mutant sequences observed for each mutant. (A) *ift172*, (B) *mks1*, (C) *tmem216*, (D) *tmem67*, (E) *rpgrip1l*, (F) *cc2d2a*, (G) *b9d2*, (H) *cep290*, (I) *tctn1*, (J) *nphp1* (ex1) and (K) *nphp1* (ex4), (L) *nphp4* (ex3) and (M) *nphp4* (ex4)**.** Various out-of-frame mutants were identified, and the mutant propagated for analysis is colored with blue. sgRNA and PAM site are indicated. Red points out nucleotides that are inserted and dash indicates nucleotides deleted.

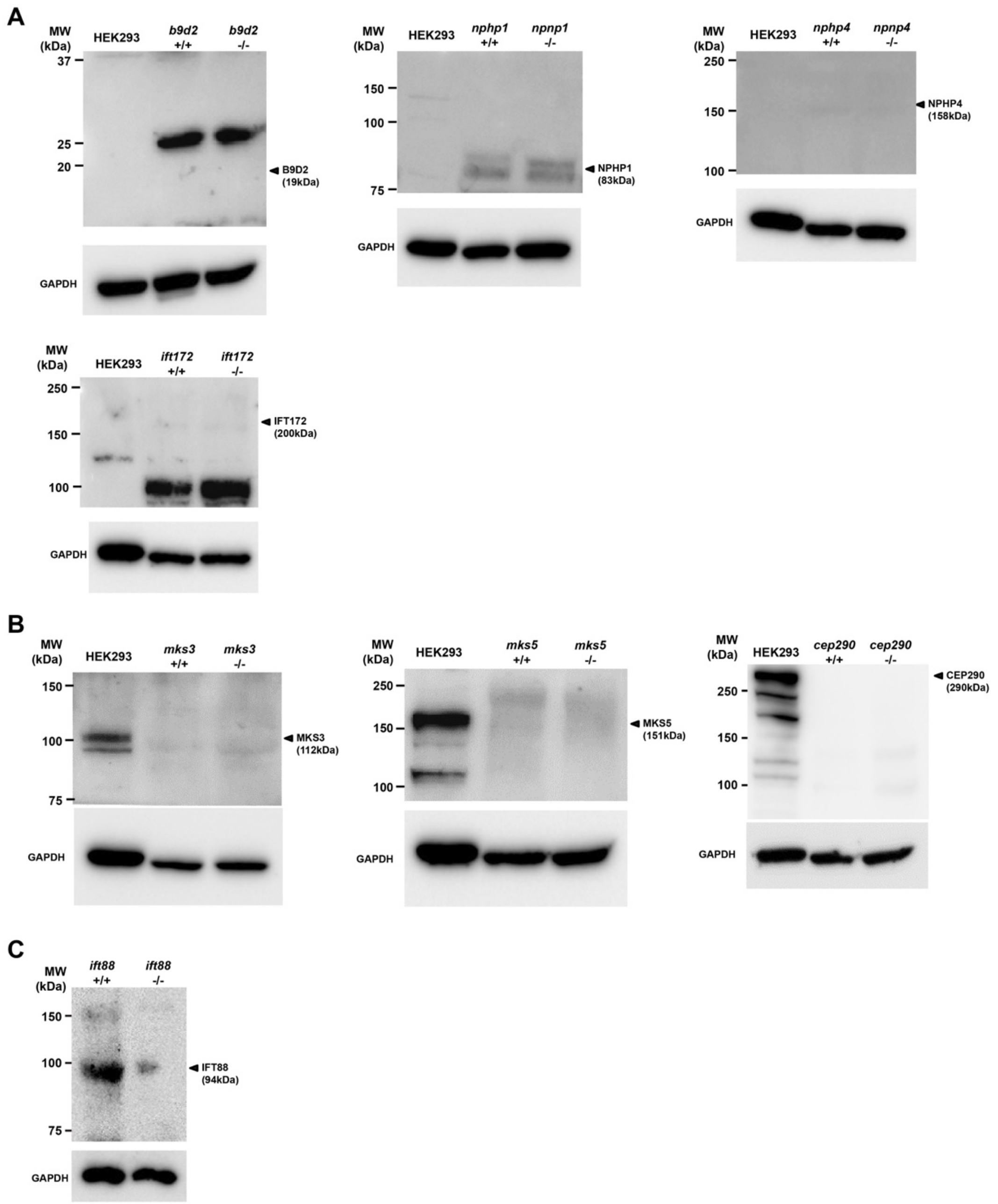

Fig. S3. Western blotting analysis to validate cilia mutants. (A) Blots demonstrate B9D2, NPHP1, NPHP4 and IFT172 antibodies do not detect human HEK293 cells or zebrafish 2 dpf embryo TZ proteins. (B) The blots demonstrate MKS3, MKS5 and CEP290 antibodies working against human HEK293 cells but not with zebrafish 2 dpf embryo proteins. (C) Blot demonstrates the loss of full-length protein in *ift88*$^{-/-}$ mutants. GAPDH was used to quantify the loaded protein amount. The arrow in each figure pointed out the anticipated band for each protein and the molecular weight of the protein was indicated.

Disease Models & Mechanisms • Supplementary information

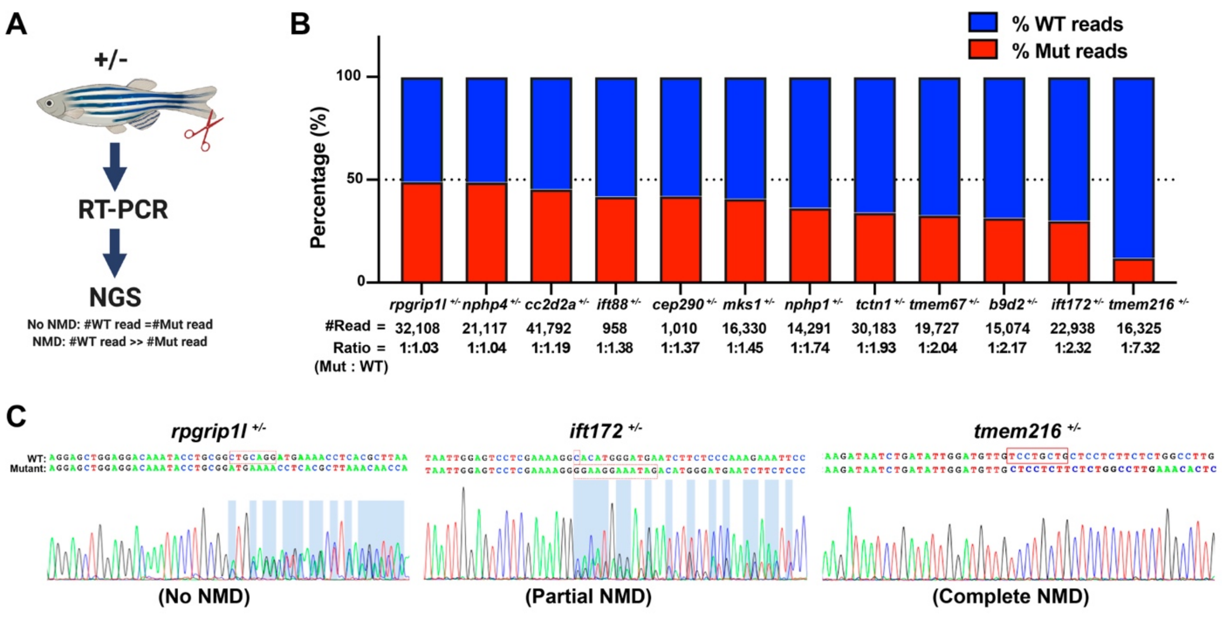

Fig. S4. Nonsense Mediated Decay (NMD) in 12 cilia-related mutants. (A) Experimental workflow to determine NMD. RNA extracted from tails of heterozygous mutants, RT-PCR, and then submitted for NGS to determine the ratio of wildtype to mutant sequences. (B) Bar graph depicting the ratio of wildtype (blue) and mutant (red) reads observed in NGS data. The total number of reads for each sample is indicated. We categorized mutants as no NMD (ratio 1:≤1.5), partial NMD (ratio 1:>1.5 but <3), strong NMD (~1:>5). (C) To validate a non-NGS alternative to determine NMD, Sanger sequencing of *ift172* (partial NMD), *tmem216* (complete NMD), and *rpgrip1l* (no NMD) is depicted. Note equivalent height of mutant and wild-type sequence in *rpgrip1l* chromatogram, half height of mutant versus wildtype in *ift172* chromatogram, and lack of mutant sequence in *tmem216* chromatogram.

Disease Models & Mechanisms • Supplementary information

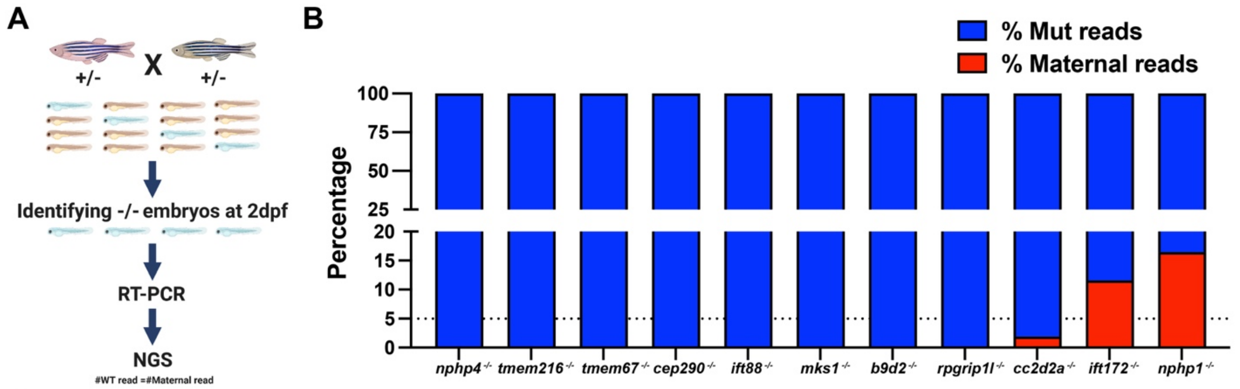

C

	NGS reads			NMD	Adj. NGS reads			MRC
	mutant sequence	wild-type sequence	total sequence	WT:Mut (mut # adj. factor)	adj. mutant sequence	wild-type sequence	adj. total sequence	WT:adj.total
nphp4$^{-/-}$	8697	0	8697	1.04	9061	0	9061	0.00%
tmem216$^{-/-}$	67775	27	67802	7.32	496113	27	496140	0.005%
tmem67$^{-/-}$	37425	13	37438	2.04	76347	13	76360	0.02%
cep290$^{-/-}$	42280	17	42297	1.37	57924	17	57941	0.03%
ift88$^{-/-}$	42439	26	42465	1.38	58566	26	58592	0.04%
mks1$^{-/-}$	27307	28	27335	1.45	39595	28	39623	0.07%
b9d2$^{-/-}$	36212	60	36272	2.17	78580	60	78640	0.08%
rpgrip1l$^{-/-}$	11076	18	11094	1.03	11458	18	11476	0.16%
cc2d2a$^{-/-}$	33526	783	34309	1.19	40047	783	40830	1.92%
ift172$^{-/-}$	1020	310	1330	2.32	2364	310	2674	11.59%
nphp1$^{-/-}$	6120	2140	8224	1.74	10664	2140	12768	16.48%

Fig. S5. The contribution of maternal RNA for cilia-related genes. (A) Workflow to determine maternal RNA contribution with RNA extracted from homozygous-mutant embryos at 2 dpf. (B) Limited maternal RNAs (wildtype sequence) were detected in mutants at 2 dpf. The percentage of mutant (blue; zygotic mRNA from -/-, and maternal null mRNA from +/- mom) or WT (red, maternal + RNA from +/- mom) reads is indicated. (C) The detailed table showing actual NGS reads and adjusted reads (based on NMD) of mutant alleles in 12 lines. adj.: adjusted; MRC: maternal mRNA contribution.

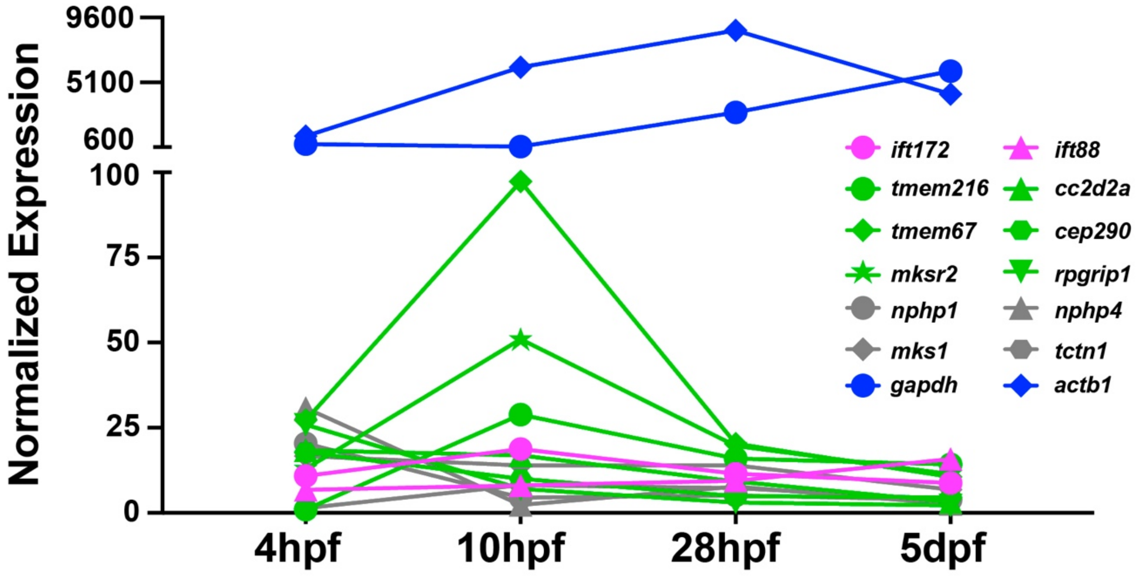

Fig. S6. Overall mRNA expression in early developmental embryos for cilia-related genes. Normalized expression of 12 cilia-related genes and two house-keeping genes including gapdh and actb1 at 4 hpf, 10 hpf, 28 hpf and 5 dpf (Chew et al., 2013). Categories of CDT phenotype were color labelled. *ift172* and *ift88* (most severe, magenta); *tmem216*, *cc2d2a*, *tmem67* and *cep290*, *b9d2* and *rpgrip1l* (green) and *mks1*, *nphp1*, *nphp4,* and *tctn1* (no CDT phenotype, grey) mutants and *gapdh* and *actb1* (housekeeping, blue).

Disease Models & Mechanisms • Supplementary information

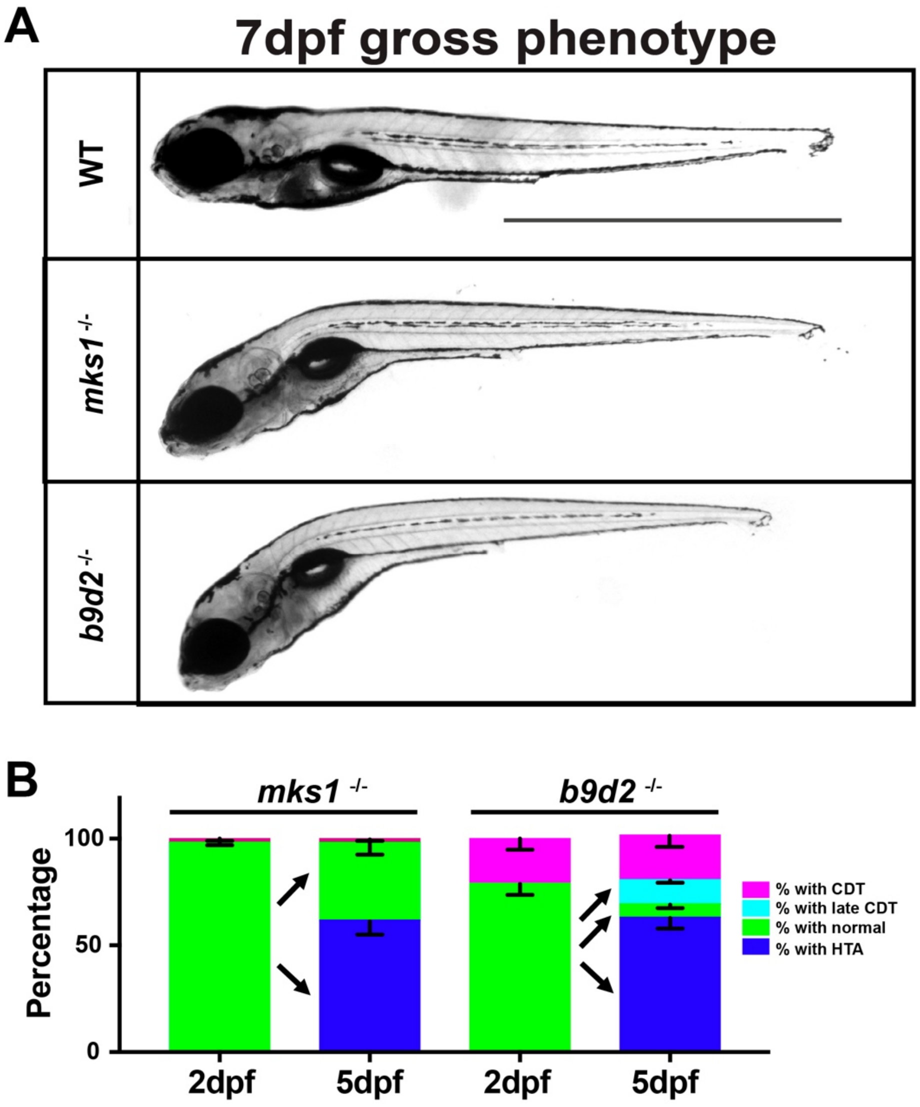

Fig. S7. Variable penetrance of curl-down tail (CDT) phenotype and decreased head-trunk angle (HTA) in *mks1* and *b9d2* mutants. (A) Lateral views of wildtype and mutants with HTA phenotype at 7 dpf. Scale bar: 2000µm. (B) Bar graph representing distribution of CDT and HTA phenotype at 2 dpf and 5 dpf. All homozygous mutant embryos were obtained from single pair of heterozygous mutant crosses that were dechorionated at 1 dpf. Embryos were sorted for CDT at 2 dpf and then reanalyzed at 5 dpf. All embryos were genotyped after phenotype analysis at 5 dpf. For *mks1*$^{-/-}$ N= 168 from 12 breeding pairs of *mks1*$^{+/-}$ adults. For *b9d2*$^{-/-}$ N= 159 from 15 breeding pairs of *b9d2*$^{+/-}$ adults. Arrows points out the derivation of the phenotypes from 2 dpf to 5 dpf. The N number in each group shown are from at least three independent experiments. Error bars represents ±SEM.

Disease Models & Mechanisms • Supplementary information

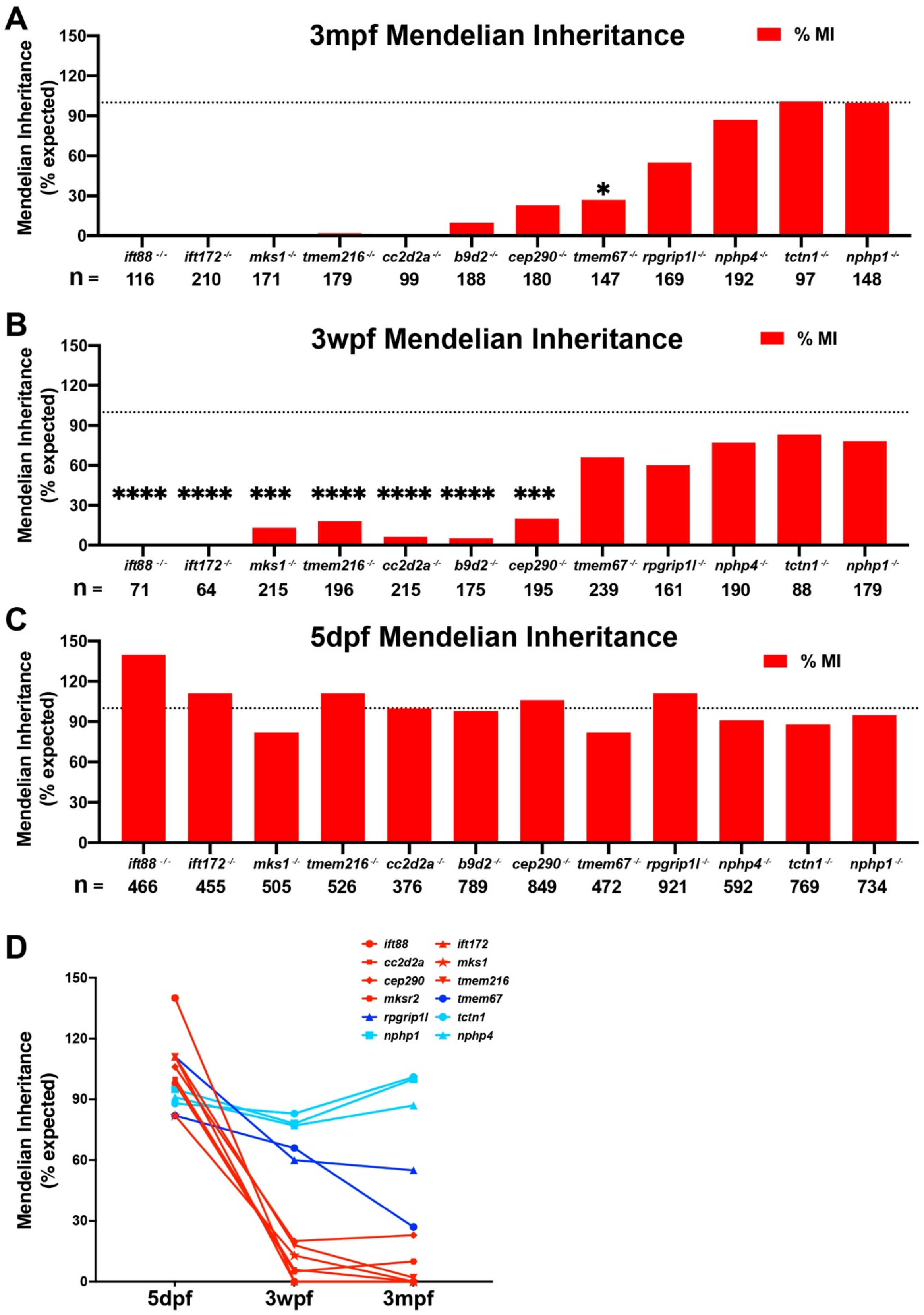

Disease Models & Mechanisms • Supplementary information

Fig. S8. Mendelian inheritance of 12 cilia-related mutants at different timepoints. Embryos were obtained from heterozygous mutant cross. (A) Mendelian Inheritance (MI) calculated as the percentage of the number observed -/- divided by the number expected -/- (1/3 of the sum of the observed +/+ and +/-) of 12 mutants at 3 mpf. Mutants were ordered based low MI to high. Fisher's exact test for mutants versus their siblings between 3 wpf and 3 mpf. *, $p < 0.05$. (B) MI at 3 wpf. Fisher's exact test for mutants versus their siblings between 3 wpf and 5 dpf. *, $p < 0.05$. ***, $p < 0.001$; ****, $p < 0.0001$. (C) MI at 5 dpf. The total number at 3 dpf, 3 wpf and 3 mpf for each mutant is indicated and from at least two independent experiments. **D.** line plot of MI for each mutant over the three timepoints.

A

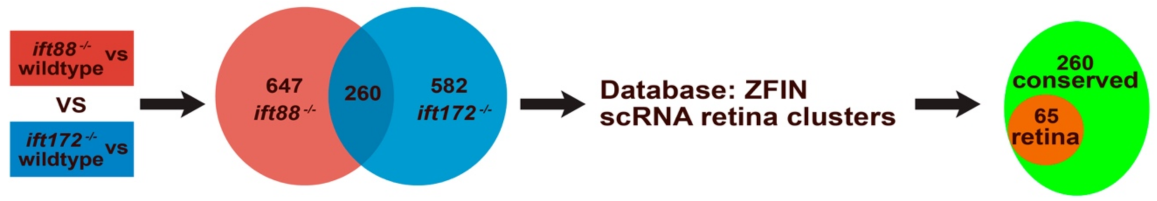

B

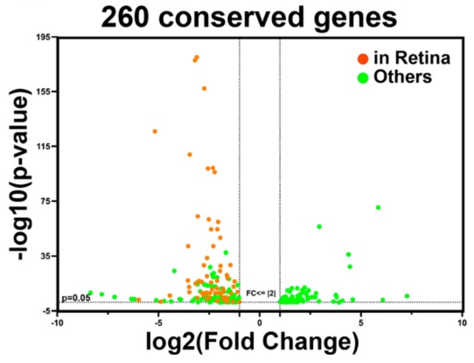

C

Retina-associated 65 genes

Biological Process	Count	$-\log_{10}$(p-Value)
visual perception	22	31.72
protein-chromophore linkage	9	11.89
signal transduction	26	11.89
*phototransduction	15	27.59
cellular response to light stimulus	8	10.00
G-protein coupled receptor signaling pathway	14	4.49
optomotor response	3	1.92
cone photoresponse recovery	2	1.80
*photoreceptor outer segment	10	16.74
queuosine biosynthetic process	2	1.57

*KEGG pathway & cellular components

Fig. S9. Transcriptional analysis of the retinal gene signatures. (A) Schematic of transcriptional analysis. First, we performed DEG analysis between *ift88* mutants and wildtype controls, as well as *ift172* mutant and wildtype controls, and then determined the conserved gene set of DEGs. This would represent common transcriptional signatures associated with common phenotypes like retinal degeneration. We manually curated the top DEGS if they are expressed in the retina, based on ZFIN database and/or published single-cell retina RNA sequencing analysis and defined a subset of 65 retinal expressed genes whose expression is altered in both IFT mutants. These genes were subsequently used in the heatmap generation in Fig. 5C. (B) Volcano plot of conserved 260 DEGs between these two datasets. Genes expressed in retina are represented with orange dots, rest are indicated with green dots. (C) G0 analysis confirmed these 65 genes are involved in retinal processes.

Disease Models & Mechanisms • Supplementary information

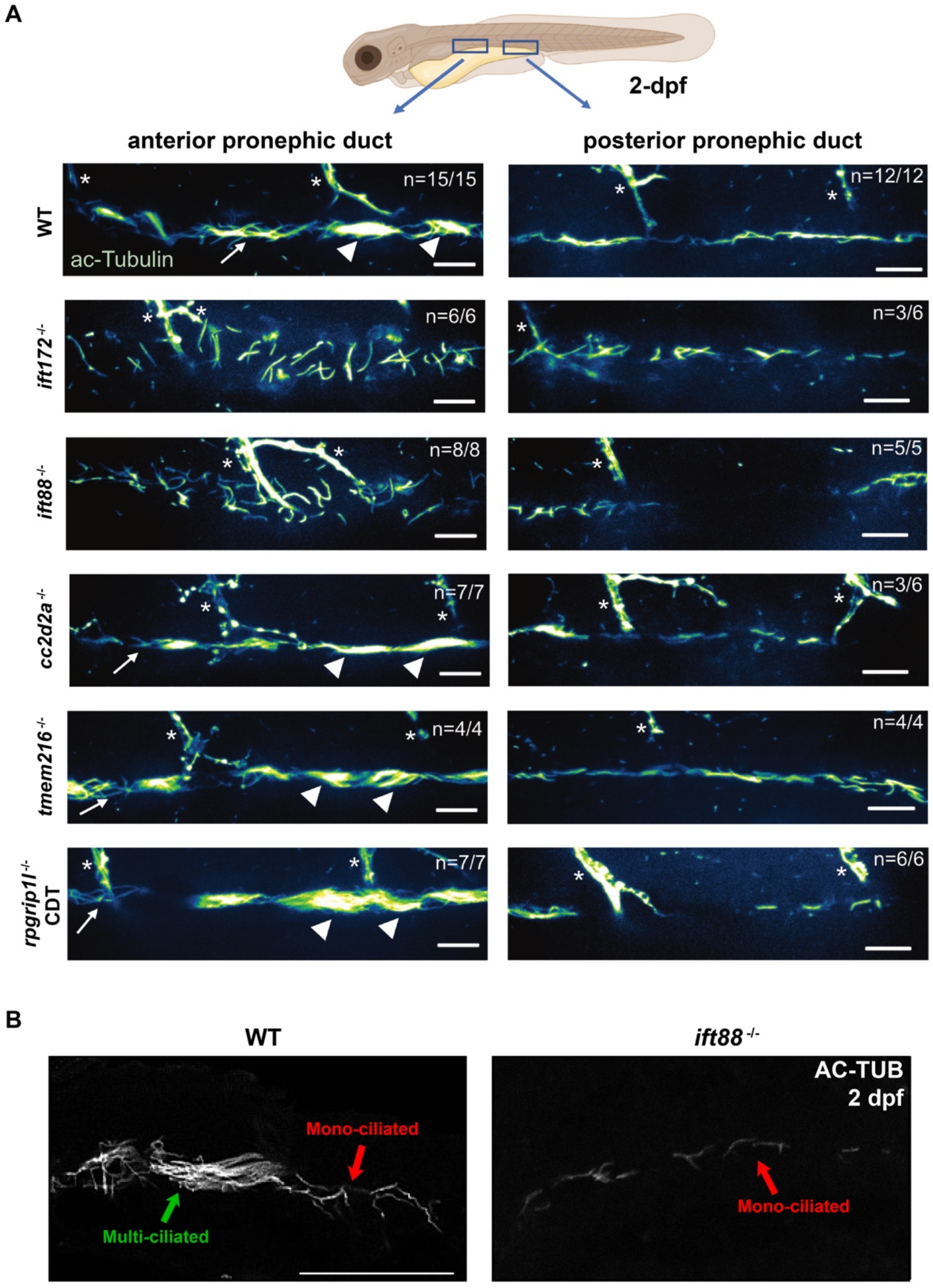

Fig. S10. Immunostaining of cilia in pronephric duct. (A) Acetylated-Tubulin antibodies were used to detect cilia in anterior and posterior regions of the pronephric duct (box shown in zebrafish icon) at 2 dpf. Representative images of anterior and posterior pronephric du ct

Disease Models & Mechanisms • Supplementary information

acetylated-tubulin staining (green fire blue LUT) are shown for wild-type, *ift172*$^{-/-}$, *ift88*$^{-/-}$, *cc2d2a*$^{-/-}$, *tmem216*$^{-/-}$ and *rpgrip1l*$^{-/-}$ embryos. Wild-type siblings were analyzed for each mutant. All mutant embryos analyzed had a curled down tail phenotype. In wild-type embryos, the anterior pronephric duct has a mixture of multiciliated tufts (arrowhead) and monocilia (arrow). Posterior pronephric ducts have only monocilia. Motor axons are also stained by acetylated-tubulin antibodies (marked with asterisks). n=number of embryos with the depicted staining/total number of embryos analyzed. Scale bar=25mm. (B) The zoom-in image of anti-acetylated-tubulin stained pronephric duct of 2 dpf wildtype (left) and *ift88*$^{-/-}$ (right) zebrafish embryos. Green arrow indicates multi-ciliated tuft, while red arrow points out single cilia. Scale bar: 50μm.

Disease Models & Mechanisms • Supplementary information

A

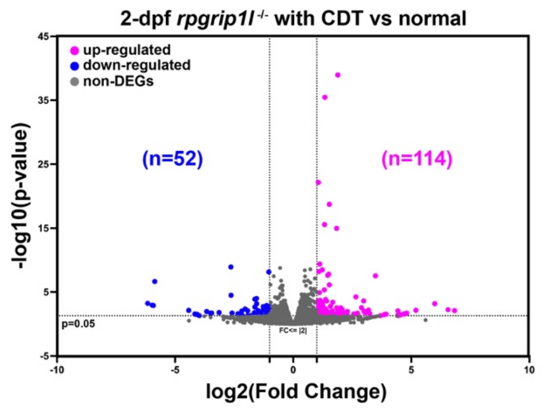

B

2-dpf *rpgrip1l*$^{-/-}$ with CDT vs normal

Gene ontology terms	Count	$-\log_{10}$(p-Value)
zinc finger, C2H2	13	3.70
Cell adhesion	5	1.64
calcium ion binding	6	0.64
nucleosome assembly	4	1.38
sensory perception of sound	2	1.02
nucleic acid binding	3	2.01
metal ion binding	20	1.72
*Regulation of actin cytoskeleton	4	1.37
*MAPK signaling pathway	4	1.21
GTP binding	6	1.00

*KEGG pathway

C

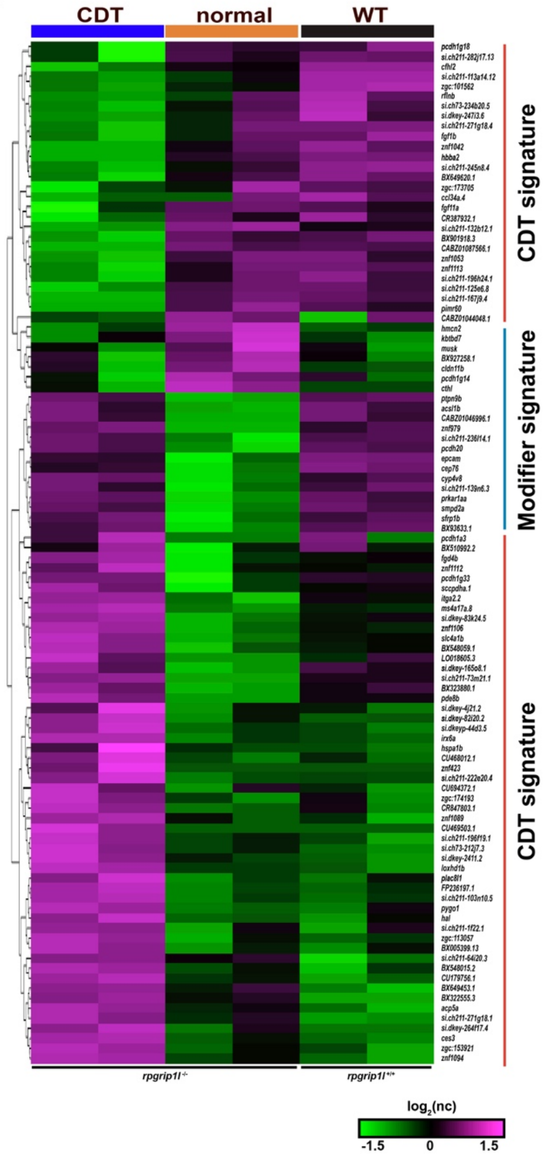

Fig. S11. Bulk RNA-Seq analysis for 2 dpf *rpgrip1l*$^{-/-}$ with curl-down tail versus with normal-looking tail. (A) Volcano plot showing 166 DEGs comparing *rpgripl1* mutants with CDT verse with normal phenotype at 2 dpf. (B) Gene ontology (GO) analysis of 166 DEGs showing at the *rpgril1l* mutants with variable correction at 2 dpf. (C) k-means clustering of log2 normalized counts for TOP 100 DEGs in 2 dpf *rpgrip1l*$^{-/-}$ with curl-down tail (CDT), *rpgrip1l*$^{-/-}$ with normal tail (normal) and *rpgrip1l*$^{+/+}$groups (WT).

Disease Models & Mechanisms • Supplementary information

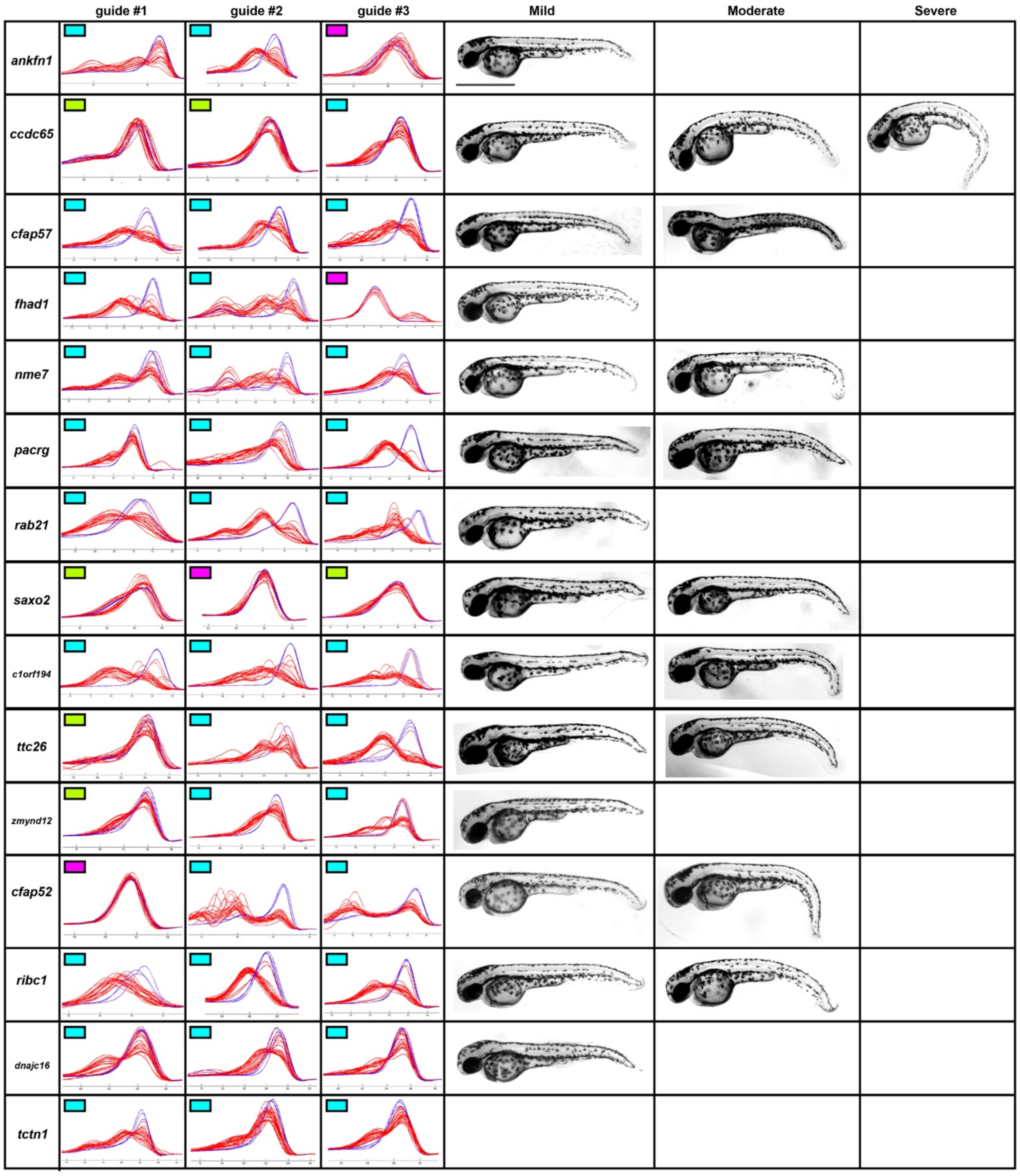

Fig. S12. Gross null phenotypes of F0 embryos targeting 16 different cilia-related gene candidates by three-guide injection. HRM curves showing the cutting efficiency of guides and lateral view images of 2 dpf injected G0 embryos of *ankfn1*, *ccdc65*, *cfap57*, *fhad1*, *nme7*, *pacry*, *rab21*, *saxo2*, *c1orf194*, *ttc26*, *zmynd12*, *cfap52*, *ribc1*, *dnajc16,* and *tctn1* genes. Representative images of observed "severe", "moderate" and "mild" curl-down tail (CDT). Scale bar: 1000µm. The gRNA efficiency was color-labelled with blue (high-efficiency), yellow (low-efficiency) and magenta (with no efficiency).

Table S1. HRM primers used for validation of cilia mutants.

HRM Genotyping Primers		
	**Forward Primer**	**Reverse Primer**
ift88 oval allele:	CAAGCAAAAGAACTATACAAAGGCTA	GGGCATTTGAAATCTGATCC
ift172 Δ1+12 allele:	GCGAGTTTTTAATTGGAGTCCT	TTGGAATTTCTTTGGGAGAAGA
mks1 +2 allele:	TTTCTCAGCAGGAGAGAGGAG	TTTGACATACAGGAGGCCGT
tmem216 Δ8 allele:	TGATACTCCCTTACCCACAAGA	GGCACTGACCATAGAAAAGTCTG
tmem67 Δ11 allele:	TGTTAAAAATAAGCGTTTTCTCCTG	TCAGAAGGCTGTCGAAATGA
rpgrip1l Δ7 allele:	GAGAGAGGCAGAACGTGTCC	CTCAAACCTGCGGATCTTGT
cc2d2a Δ7 allele:	GGATCCAGCCAATCAGAAAA	TGCATGCTGTGTTTTTCTCTG
b9d2 +16 allele:	TTGCATATCATTGGACAGATCA	AAAGGCTGTTCTGTGGGAAA
cep290 Δ23 allele:	AAACATTTTCTGCGGGATGA	GCTGCCTACCTCTTCATTGG
tctn1 +5 allele	TGAGCAGTGCAACTGATGTG	TCAAACTTTACCCGGTATCCTG
nphp1 +2 allele:	GGGAAGAGGAGAAGCGGTTA	TGAGAGTGAATGTGTGTTGGAA
nphp4 Δ7 allele:	AACCATGTTTCTTTTTAGGCTGA	GGCTAACAGTGCGGTCAGAG

Table S2. Primers used for examining NMD and maternal RNA for cilia mutants.

Primers to detect NMD and Maternal RNA		
	**Forward Primer**	**Reverse Primer**
ift88 oval allele:	NA	NA
ift172 Δ1+12 allele:	AGGGCAAACTGCTGACTCAT	GTGCCAGTGGACAAATTCCT
mks1 +2 allele:	CCCTGTCTGAACACCTCCAT	AGGTGTAGGCTGAGCTGGAA
tmem216 Δ8 allele:	CGATGTTTGTCTCCGCCTAC	GAGCACAGCATTGAGGACAA
tmem67 Δ11 allele:	TCAAGCGTCATCTGATCCTG	AGGCAACTGAGGTAGGCAGA
rpgrip1l Δ7 allele:	TCTCGTGCTGATGAAACTGC	CTGAGAGCTGCTTTCGGAGT
cc2d2a Δ7 allele:	GAGATGGCTGGTGGAAAAGA	CTGGCAGCAAAAGAGGAGTC
b9d2 +16 allele:	GACAGGAAGGCGCTGTAAAC	GGCGAAGAATAATGCAGAGC
cep290 Δ23 allele:	GTCAAACCACGGGACCTAAA	TTGGCTTTGGTGAGTCTCCT
tctn1 +5 allele	CCTCGCTCTACACCGAAGAC	ACAATCACGTGCCAAATCAA
nphp1 +2 allele:	AAACAAAGAGGCCTTGCAGA	AATGCTGAGATCCCCTTCCT
nphp4 Δ7 allele:	AAGCAGTGGGGTCAGGTTTT	CCATTATGAACCCCAACCAG

Table S3. IDT guide RNAs used to target 16 different cilia-related candidate genes.

Gene	Guide Sequence	PAM	Exon
Ift172	AACCTATGTGGTCAAGTCTA	TGG	3
	GGAGTCCTCGAAAAGGCACA	TGG	9
	CAAGCAAGTGGCCTACCTGT	GGG	30
cc2d2a	AGTGTCTGAGTGTCTCACCA	GGG	3
	GAAGTTGTAGGCCTCTTCGG	CGG	8
	TCCACAAACGGCCTGATCAG	AGG	26
p53	CCAAGAGTTCGCGGAGCTCT	GGG	2
	CCGGAGACAAGCGACTATCC	CGG	4
	CGTCCACCACCATTTGAACG	GGG	5
puma	AGAGTGGACGAACATAACTC	TGG	2
	CTGGACGCTGTCCAAGCTGA	AGG	2
	GCTGGACGTGGACTCCTCTG	AGG	3
tctn1	ACTGATGTGAGAAGTTTACC	AGG	2
	ACTCTAGGAACTCTGAGCTT	GGG	4
	TCACCAGACTGCAAGAGCAT	AGG	6
lbx1a	GGTGAGAAGCATCCGTCCAC	GGG	1
	GGTGGAGGAAGATGGTCCAA	CGG	1
	TCTTCTCAAGTTCGTAGATT	TGG	2
	AAGTTCGTAGATTTGGTGAT	TGG	2
ptk7a	GGTTTATGGACGAGAAGACG	CGG	1
	TGTGAAGTAAACGACCCTCA	GGG	2
	AACCCCAGGACGCTCTGCAC	GGG	2
	TGAATCTCTGCCACTGACTC	AGG	3
b9d2	CATATCATTGGACAGATCAT	TGG	2
	GGACAGATCATTGGGGCCAC	CGG	2
	TGATTTGCACTACACTACTA	AGG	3
	GTAGTGTAGTGCAAATCAAT	GGG	3
ankfn1	AGGGAGCCACTCATCCTTCG	AGG	1
	GTCATGATGGAGATGTCCAG	AGG	2
	CACCTGGTTGGACAGTTCAC	TGG	3
ccdc65	CAACCCGGCCAGTTTTCCAC	CGG	1
	CAACTTCAAGAGCAGTCTAG	TGG	3
	ATCACTGTAGTGCTTTTCCA	TGG	4
cfap52	ATAATAACAGTGCAGCCAAG	AGG	2
	ACTCAAGTTGGTGTACTCTA	AGG	4
	GTGGAACATCGAAAGTAAGG	AGG	4
cfap57	GTTTCCACACGGAAATATGA	TGG	1
	CCAATCAGATACTTTGAGTC	AGG	2
	CTTACGATGGCTGTAACCTG	TGG	4
dnajc16	TGATCAAGATAACCTCAGAT	TGG	3
	TTGACGACACCAAGGATGGA	TGG	4
	CAGAAGGCACTTGGTCAAAC	AGG	5
fhad1	TGGAGTTTAAATACCCAGTT	TGG	1
	CCAAAGTGAAGCTGGTCTCC	TGG	2

Disease Models & Mechanisms • Supplementary information

	AATATCATGCCACTATTGAC	TGG	2
nme7	TGAGTGGTACGACCCCAGTG	CGG	2
	CAACAAGCTGGGCAGTAAGA	AGG	3
	TGTGCCACACCAGAATCCGT	CGG	6
Pacrg	TCCTTTAGCCAAAGGTTCAA	AGG	1
	ACGTTCATAAAACTTCCGGA	AGG	2
	CATGACATGCTGGAGCACGG	CGG	3
rab21	GAAGACGTACTCGTTCAAGG	TGG	1
	TCTGTAGTAGATGGGCCCAA	GGG	3
	GGTGTACGACGTCACAGATG	AGG	3
ribc1	CAGGTCCACGCCAATAACCC	GGG	1
	ACATTAATTATGACCGAAAG	GGG	2
	AAACCTGAGAGCTCAAAAGG	AGG	3
saxo2	CTGACAACACGTTGGTCTGA	GGG	2
	CAAGTCTGGAGAGATTGATT	TGG	3
	TGGGAATTCCACAACCTTCC	AGG	4
c1orf194	CACAAAATGATGAACCGTGG	AGG	2
	TAATCCAAGATGATGATCGT	AGG	3
	TGAATGTCCAAACTGTCCTT	CGG	3
ttc26	TTAGATAATCCTCAAGCCTT	GGG	2
	GAGCACGCAGATCTTTGGAC	TGG	3
	ACAATCCGGCCTCAAAGTGA	GGG	4
zmynd12	TCTCCTTTAGGATTTGCTAG	CGG	1
	ACAAGCTTTTTCATGAATAC	TGG	2
	CGGATCATGTACTGTCGAAC	TGG	3

Table S4. DEGs for 5 dpf conserved genes between *ift172*$^{-/-}$ versus *ift172*$^{+/+}$ and *ift88*$^{-/-}$ versus *ift88*$^{+/+}$.

Click here to download Table S4

Table S5. DEGs for 2 dpf *rpgrip1l*$^{-/-}$ with curled-down tail versus *rpgrip1l*$^{-/-}$ with normal-looking tail.

Click here to download Table S5

Disease Models & Mechanisms • Supplementary information

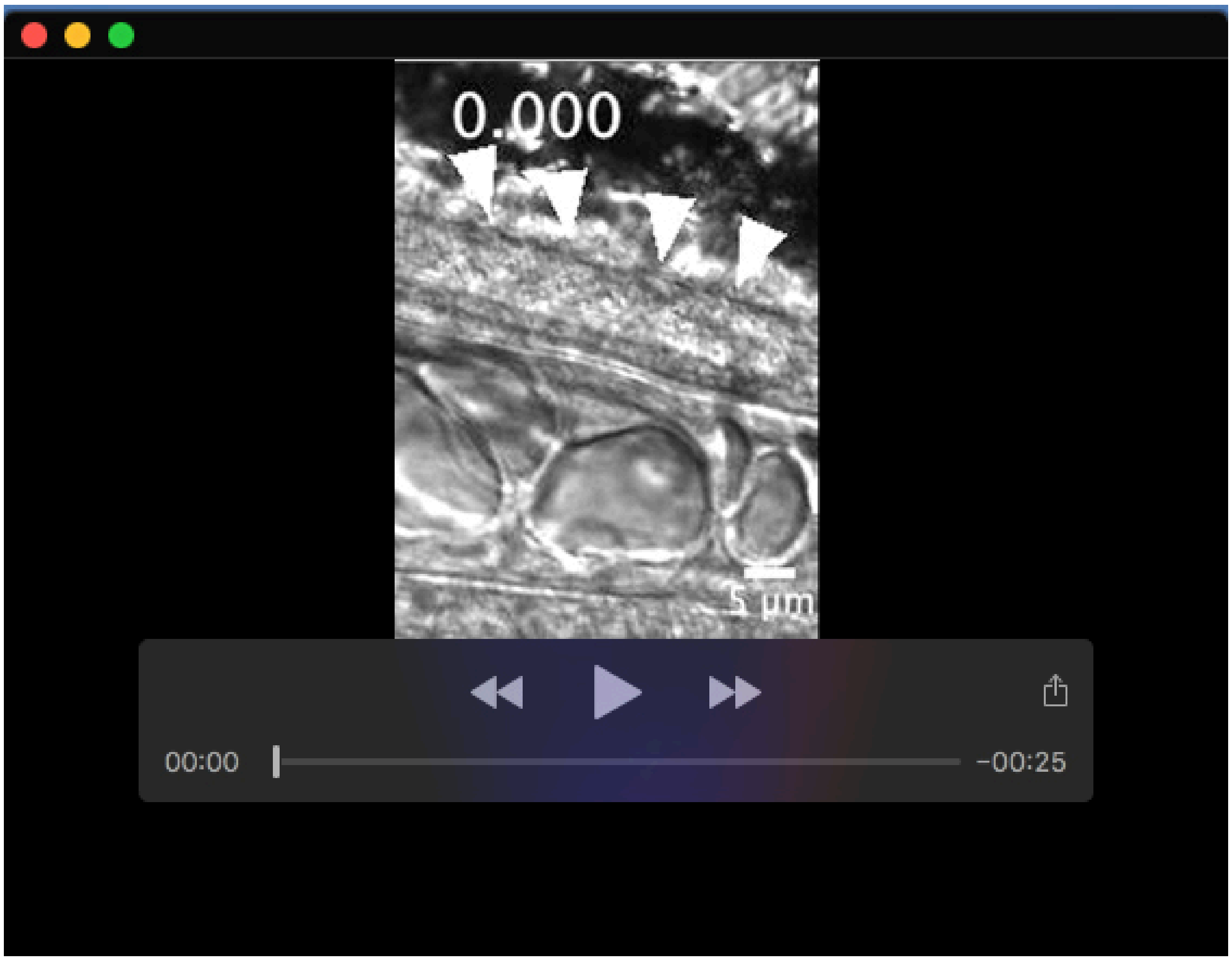

Movie 1. Motile cilia in the posterior neural tube of a 2 dpf wild-type (cc2d2a sibling) embryo. Arrowheads point to the neural tube. Differential interference contrast images were captured at 60 frames per second and playback speed is real time. Timestamp=seconds. n=6/6 (number of wild-type embryos with motile neural tube cilia/number of embryos analyzed).

Disease Models & Mechanisms • Supplementary information

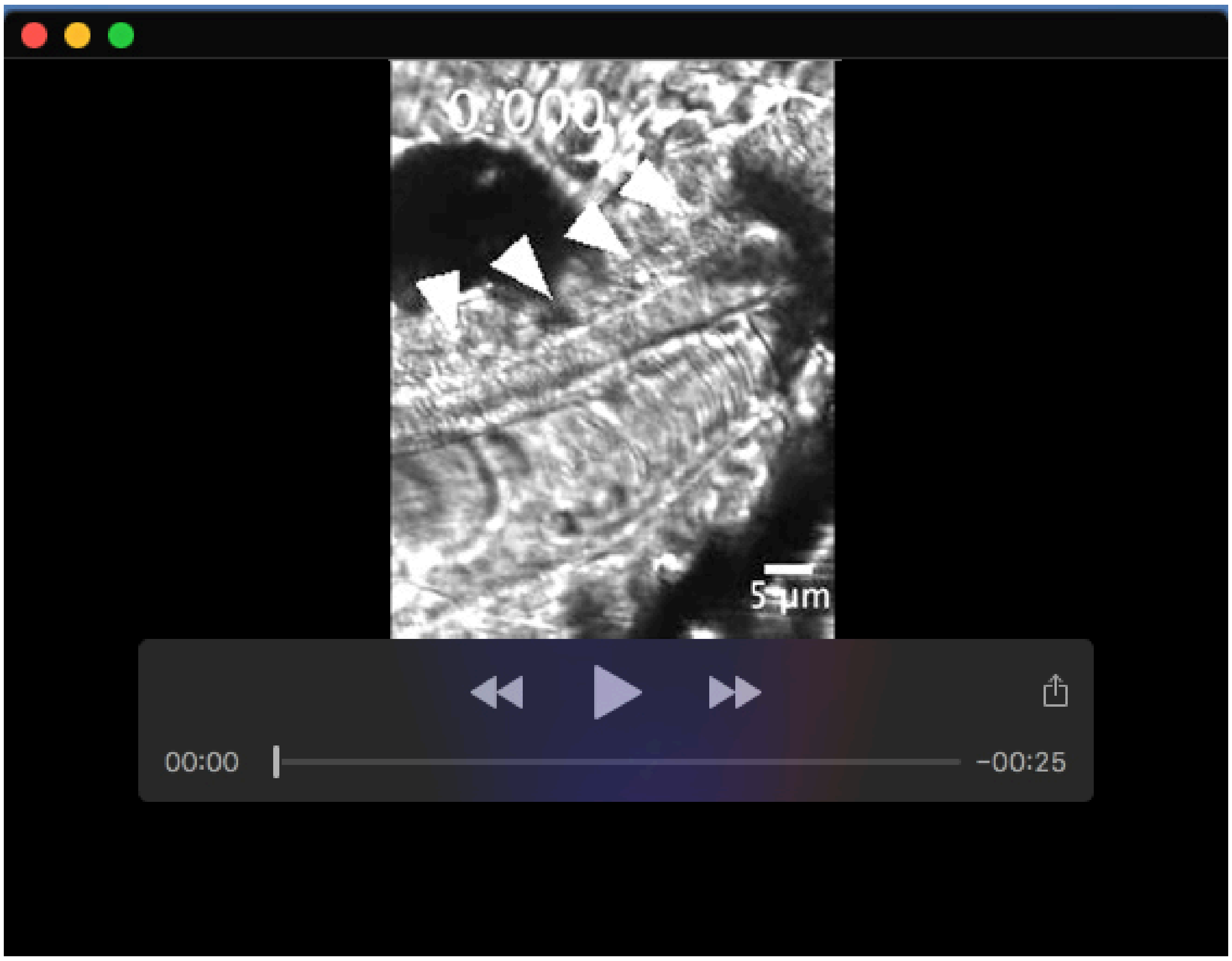

Movie 2. Motile cilia in the posterior neural tube of a 2 dpf cc2d2a -/- embryo with a curled down tail. Arrowheads point to the neural tube. Differential interference contrast images were captured at 60 frames per second and playback speed is real time. Timestamp=seconds. n=7/7 (number of mks6 mutant embryos with motile neural tube cilia/number of embryos analyzed).

Disease Models & Mechanisms • Supplementary information
